# An exploratory, cross-cultural study on perception of putative cyclical changes in facial fertility cues

**DOI:** 10.1038/s41598-021-96454-w

**Published:** 2021-08-19

**Authors:** Urszula M. Marcinkowska, Benedict C. Jones, Huaijan Cai, Jorge Contreras-Garduno, Ike E. Onyishi, Charles T. Orjiakor, Keshav Prasai, Farid Pazhoohi, Hirokazu Taniguchi, Anthony J. Lee

**Affiliations:** 1 Department of Health Sciences, Jagiellonian University Medical College, Cracow, Poland; 2 School of Psychology, University of Strathclyde, Glasgow, Scotland, UK; 3 Institute of Psychology, Chinese Academy of Sciences, Beijing, People’s Republic of China; 4 ENES Campus Morelia, National Autonomous University of Mexico, Morelia, Mexico; 5 Department of Psychology, University of Nigeria, Nsukka, Nigeria; 6 Uniglobe H.S.S/College, Kathmandu, Nepal; 7 Department of Psychology, University of British Columbia, Vancouver, Canada; 8 Shimonoseki City University, Shimonoseki, Japan; 9 Division of Psychology, University of Stirling, Stirling, Scotland, UK

**Keywords:** Evolution, Psychology

## Abstract

Although many researchers have argued that facial traits evolved as honest cues to women’s current fertility (possibly via changes in facial femininity), evidence that women’s facial attractiveness is significantly, positively related to probability of conception throughout menstrual cycle is mixed. These mixed results could reflect differences among studies in the methods used to assess facial attractiveness (i.e., forced choice versus rating-scale methods), differences in how fertility was assessed, differences in perceiver characteristics (e.g., their own attractiveness), and facial preferences possibly being moderated by the characteristics of the living environment. Consequently, the current study investigated the putative effect of cyclical changes in fertility on women’s facial attractiveness and femininity (1) using forced choice and rating-scale method, (2) conducting both ovulation tests and repeated daily measures of estradiol assessing the conception probability, (3) based on a culturally diverse sample of perceivers, while (4) controlling for inter-individual variation. Although we found some limited evidence that women’s faces became more attractive when conception probability increased, these effects differed depending on the methods used to assess both attractiveness and fertility. Moreover, where statistically significant effects were observed, the effect sizes were extremely small. Similarly, there was little robust evidence that perceivers’ characteristics reliably predicted preferences for fertility cues. Collectively, these results suggest that mixed results in previous studies examining cyclical fluctuation in women’s facial attractiveness are unlikely to reflect inter-cultural differences and are more likely to reflect differences in the methods used to assess facial attractiveness and fertility.

## Introduction

Facial cognition is of great importance for human biology studies, and has potential implications for resources acquisition, social interactions, and mating^1^. Identifying factors that affect facial cognition can provide insights into mechanisms underpinning important social outcomes and behaviours^2^. Women’s sexual dimorphism displayed in secondary sexual characteristics (e.g., facial femininity) may signal to men their reproductive potential^3^,with variability in sex hormones levels, such as estradiol and progesterone, having been identified as a putative underpinning mechanism for this association^4^. However, previous research on men’s preference for facial femininity, or associations with underlying sex hormones, have provided mixed results. A potential source of these mixed findings may be due to unmeasured inter-populational and inter-individual differences or varying methodological approaches.

### Facial cues to fertility

Previous studies have suggested a correlation between changes in conception probability throughout the menstrual cycle and women’s facial attractiveness^4,5^. This could suggest that sexual selective pressure may favour men that are able to detect increases in conception probability based on discrete changes in facial cues^6^. However, while some studies support that cognition is attuned for detecting subtle cues of changes in fertility^5,7^, other studies have not found this association^8,9^. A similar lack of congruent results has been observed regarding effects of sex hormones on facial appearance. Levels of sex hormones (namely estradiol and progesterone) fluctuate throughout menstrual cycle, and these pre-defined fluctuations are necessary for a successful conception^10^. Echoing the mixed results of studies based on timing within menstrual cycle, some studies have found that women’s facial attractiveness does track changes in levels of sex hormones^11^, while other studies did not support this hypothesis^12^.

### Interpopulation differences in perception of attractiveness

Although femininity, due to its purported relation to fertility, is highly valued among virtually all measured cultures^13^, the strength of the preference for women’s sexual dimorphism varies according to environments’ characteristics. It is not yet clear which specific environmental cues are related to the preferences for increased sexual dimorphism. Some evidence has been reported for a link between preferences for sexual dimorphism and changes in harshness of the environmental conditions^14^, income inequality and homicide rate^15^, national health indices^16,17^, and the urbanization and developmental rate of the population^18^. However, most of these studies investigate women’s preference for sexual dimorphism in male faces—comparatively little work has investigated potential cross-national effects on preference for facial femininity in women.

### Inter-individual differences in perception of attractiveness

Aside from interpopulation variation, individual differences in the person expressing the preference also can account for variation in facial preferences. A multitude of characteristics have been found to influence human facial preferences, including age (Marcinkowska et al. 2017), sexual openness (Marcinkowska et al. 2020), relationship status and resource availability (Lyons et al. 2016), self-rated attractiveness and health^19^, contraception use (^20^ however see^21^ for null effect of hormonal contraception on sexual dimorphism preference), and family constitution, including parenthood status^22^ or similarity of judged face to self, sibling or parent^23,24^. This vast array of confounding variables putatively leads to discrepant results, depending on how the study is being conducted, and how many of possibly confounding variables have been included in the model.

### Does variation in methodological approaches account for results discrepancy

It is possible that the lack of the consistency in results of facial correlates of fertility studies is rooted in differences in methodological approaches between studies. A growing body of evidence suggests that the strength of preferences for putative fertility cues is moderated by the type of task used in measuring the preference, for example, between a forced choice and a rating task^25^. To date, little work has tested for possible associations between environmental conditions and preferences for putative facial fertility cues using both forced choice and rating scale paradigms to account for this methodological issue.

Additional confounding factor leading to incongruent results could be the differences in gauging conception probability. Previous studies have employed an array of methods, from (now strongly discouraged^26^) forward and backward counting days method, luteinizing hormone-based ovulation tests (LH tests), one-point daily hormonal measurements or multiple hormonal measurements. Recent research suggests that combination of LH tests and multiple hormone measures of estradiol may be optimal^27^.

### Aim of the current study

Based on the greatly inconsistent results of previous studies investigating facial cues to fertility, we conducted a large-scale, cross-cultural study that investigated possible sources of interindividual and interpopulation differences in perception of putative cues to fertility. This included investigating whether participant age, sex, self-rated attractiveness, self-rated health, self-rated financial difficulties, and sexual openness, as well as ecological factors such as country health/development, or inequality influence facial preferences or ratings of facial femininity. Simultaneously, we accounted for an array of methodological issues in both defining fertility status, and in how facial preference was measured. The current study employs five diverse approaches to measuring current fertility: (1) comparison of photographs of women taken in three moments varying in conception probability, (2) comparison of a subset of photographs from three distinct moments in the cycle among women who had greater chance of conceiving (based on LH-tests and estradiol levels), (3) 3-point hormonal daily measurements of estradiol, (4) 3-point hormonal daily measurements of progesterone, and (5) 3-point daily ratio of estradiol to progesterone. We also compare preferences measured using a 3-Alternative Forced Choice task (3AFC), or preferences measured on a rating scale. Given the complexity of the study and the multitude of research questions, we use an exploratory approach, i.e. conduct statistical analyses with no strong a priori predictions with the aim of identifying possible effects that future research could investigate. As such, results should be interpreted cautiously.

## Materials and methods

### Facial stimuli creation

Photographs of 88women between 18 and 36 years of age were taken three times throughout one menstrual cycle based on a sample described in^27^. All photographs were taken on a white background with Canon EOS 700 D camera with 50 mm objective and Meke Flashgun FC 100 circular ring LED external flash light. Participants were asked to stand still and look straight ahead. Height of the camera was adjusted to the height of the participant, distance between participant and the camera was kept standard. All participants were White, between 18 and 36 years of age and living in Małopolska region in Poland.

The first photograph was taken during early follicular phase, on average on the 5th day of the cycle (SD = 2.21 days). The second photograph was taken around ovulation time, on average 12 days before the onset of the next cycle (SD = 3.65 days), and 1.5 days after obtaining positive results of the LH test (SD = 1.71 days). If a participant did not record a positive result of the LH test, the second photograph was taken on the 20th day of the cycle. The third photograph was taken on average4 days before the onset of the next menses (SD = 3.54 days).

For establishing the peri-ovulatory timing of the second photograph, ovulation detection was based on two independent measures: (1) LH-based ovulation tests administered starting from 10th day of the cycle, and (2) post-hoc daily salivary estradiol (E) measurement as the highest drop of E within a cycle is a robust measure of ovulation^10^. The post-collection estradiol measurement coupled with LH test results were used for creating the so-called “textbook” group—the subset of women where the probability that the cycle during which the photographs were taken was ovulatory was higher, than in the overall sample, due to occurrence of both LH peak and E drop.

Based on the gathered photographs and menstrual hormonal data, 5 sets of face prototypes were created using Psychomorph software^28^. By averaging multiple faces together, any consistent differences in women’s facial characteristics related to conception probability/hormonal profile can be assessed, while removing information associated with individual identity.

**Figure 1:**
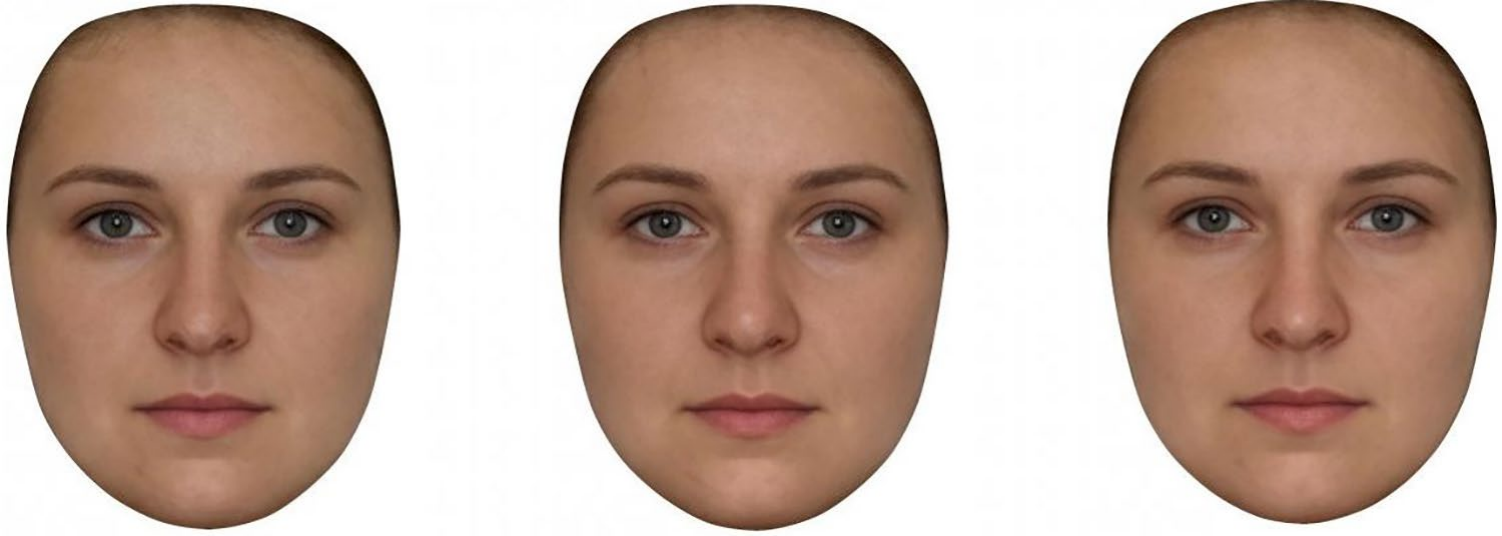
Example of visual stimuli presented to participants in the 3AFC block examining the perceived attractiveness of the “textbook” group. Left: early follicular phase, middle: peri-ovulatory phase, right: mid-luteal phase.

The first set of prototypes was created using photographs of all 88 women. Three facial composites were created based on medium, high and low conception probability. This was done by averaging the shape, colour, and texture information for all 88 facial images obtained during the early follicular, peri-ovulatory and mid-luteal phase respectively. The second set of prototypes was created using a subset of 45 images that only included women who met our strict criteria for showing “textbook” menstrual cycles (Fig. 1). As with the previous set, images of women in the early follicular, peri-ovulatory, and mid-luteal phase were used to create prototypes of medium, high and low conception probability respectively.

We created three further sets of face prototypes based on sex hormone measurements—this included an E, P, and an E/P set. To create the low E prototype, shape, colour, and texture information was average for the images of each of the 88 women’s photographs with the lowest level of E. Similarly, a high E prototype was created by averaging the shape, colour, and texture information for the each of the 88 women’s photographs with the highest level of E. The remaining images were averaged to create a medium E prototype. The same process was used to create the low, high, and medium composites for P, and E/P, except using measurements of progesterone and E/P ratio.

### Participants and procedure

Ethics approval was given by the Jagiellonian University Medical College Ethics Board (number: KBET/250/B/2014). The study was conducted in compliance with national legislation and the Code of Ethical Principles for Medical Research Involving Human Subjects of the World Medical Association (Declaration of Helsinki). The visual stimuli created for the purpose of the study is an averaged morph of many women. No individual faces are being published. Morphing technique using Psychomorph Programme (Tiddemeann et al. 2011) allows for presenting anonymous facial visual stimuli while simultaneously retaining features of interest. The process used in digitally creating the facial images used in this current study mean that the final image cannot be traced to a particular individual. As the presented faces are digitally created, no individual faces can be recognised from it and so no consent was needed for the pictures. Participants were recruited via online platforms, social media and personal communication. A total of 1606 participants (718 women, 876 men, 12 other or missing data; mean age = 29.80 years, SD = 10.10 years) were recruited from 47 countries. In all countries but Nigeria, questionnaires were completed online. The study was conducted with the understanding and written, informed consent from each participant. Data Use Ontology (DUO)-compatible consent language was adopted into informed consent. Data access conditions were clearly communicated to participants. All participants were asked to complete a short socio-demographic survey (including questions on gender, sexual orientation and age, self-rated health and attractiveness and socio-economic status), followed by Sociosexual Orientation Revised questionnaire^29^ and the block of questions on facial preferences. Within this block participants answered questions about two different characteristics of the presented facial composite: attractiveness and femininity. Questions were 3-Alternative Forced Choice (3AFC, “Please choose the face that you think is the most attractive/feminine”) or 7-point Likert scale (“On a 1–7 point scale, how attractive/feminine is the presented face?”). For the 3AFC tasks, 3 facial images were presented simultaneously on one slide with location of the faces and order of the trials being randomised between participants. For the rating task, one facial image was presented on one slide and order of slides was randomised between ratters.

As sexual orientation of the ratter can influence judgements of facial attractiveness^30^, participants who identified as predominantly homosexual on the Kinsey scale (responses ranging from 4 to 6; 144 participants) or did not identify as either male or female (3 participants) were excluded from analyses. Following Lee et al.^31^, analyses were also restricted to participants in countries where there were at least 10 participants in the sample. These criteria resulted in a final sample of 1371 participants (594 women, 777 men, mean age = 29.59 years, SD = 9.72 years) from 12 countries (Australia, China, Germany, Iran, Japan, Mexico, Nepal, Nigeria, Poland, Spain, United Kingdom, and United States of America). For detailed data preparation, see ESM1.

### Statistical analysis

For all analyses, outliers on continuous variables were winsorised to ± 3 SDs. To maximise statistical power, participants with missing data were only removed for the analyses in which missing values appeared. For the 3-Alternative Forced Choice task, data was analysed either using a Monte Carlo randomisation method^32^, or using cumulative link mixed effects models using the clmm function in the ordinal package in R^33^, which allows for analysis of ordinal outcome variables. For the rating task, data was analysed using linear mixed effect models, using the lme4^34^ and lmerTest^35^ packages in R. Continuous variables were standardised at the appropriate level (i.e., individual differences were standardised at the participant level, while country factors were standardised at the country level), while categorical variables were effect coded. Where appropriate, random intercepts were specified for participant and country. Random slopes were specified maximally following^36,37^. Given the exploratory nature of the analyses, we aimed to be thorough in the number of models conducted. We provide a description of the key results across the models here in text. For full model results, see the ESM2.

## Results

### Are judgements of attractiveness and femininity associated with putative fertility cues?

#### Three-alternative forced choice task

To assess whether choices on the 3-Alternative Forced Choice (3AFC) tasks were non-random, we followed procedures in Scott et al. (2014) using a Monte Carlo randomisation method^32^. For each judgement (either attractiveness or femininity for each of the five sets of face prototypes), we simulated 10,000 samples with sample sizes matching those of the data, where simulated participants chose one of the three faces at random. We then calculated a vector from the centroid, where the centroid represents equal choices to all three images. A larger vector represents greater evidence for non-random choice. The proportion of vectors from the simulated samples that are larger in magnitude to the observed vector from the data represents the *p*-value for the null hypothesis (i.e., shows greater evidence of non-random choice regardless of direction). Proportions of choices are shown in Table1 with visualisations included in the ESM2.

**Table 1:** Proportion of choices for each judgement, including attractiveness and femininity choices for each of the five fertility cues in the 3AFC task (non-random choices in bold).

	Low (%)	Medium (%)	High (%)	*p*-value
**Attractiveness**				
Conception probability in all cycles	33.03	32.88	34.09	.847
**Conception probability in “textbook” cycles**	**29.64**	**31.01**	**39.35**	** < .001**
E	35.20	32.25	32.55	.352
P	34.07	34.60	31.34	.296
**E/P**	**36.09**	**30.17**	**33.74**	**.030**
**Femininity**				
Conception probability in all cycles	34.72	32.64	32.64	.575
**Conception probability in “textbook” cycles**	**30.51**	**33.36**	**36.13**	**.047**
E	33.44	34.90	31.67	.354
P	34.72	33.03	32.26	.533
**E/P**	**37.41**	**30.56**	**32.02**	**.007**

For the face prototypes based on images of women with “textbook” menstrual cycles, the prototype with high conception probability (peri-ovulatory prototype) was chosen as the more attractive and more feminine significantly more often than would be expected by chance alone. For the face prototypes based on E/P ratio, the low E/P prototype was chosen as the more attractive and more feminine significantly more often than would be expected by chance alone. No other tests showed non-random choices (i.e., no statistically significant effects of fertility or hormone levels on either attractiveness or femininity judgments appeared).

### Seven-point Likert scale

For the rating tasks, we ran a linear mixed effects model for each judgement, where level of each fertility cue (low vs. high) predicted ratings of attractiveness or femininity. Participant age and sex were included in the model as covariates. High E was associated with higher attractiveness and femininity ratings. None of the other models showed significant effects of either fertility or hormone levels (see Table 2).

**Table 2:** Associations between level of fertility cue and ratings of attractiveness and femininity for each of the separate models (statistically significant differences in bold).

	Estimate (SE)	*p*
**Attractiveness**		
Conception probability in all cycles	− .04 (.03)	.245
Conception probability in “textbook” cycles	.04 (.05)	.472
**E**	**.11 (.03)**	** < .001**
P	.05 (.04)	.272
E/P	.05 (.03)	.109
**Femininity**		
Conception probability in all cycles	− .05 (.06)	.416
Conception probability in “textbook” cycles	.03 (.06)	.697
**E**	**.09 (.03)**	**.011**
P	− .11 (.07)	.156
E/P	− .03 (.04)	.479

#### Are judgements of attractiveness and femininity for putative fertility cues consistent between tasks?

For each participant, we calculated a difference score from the ratings, where the rating given to the low fertility face was subtracted from the rating given to the high fertility face. As such, larger, positive difference scores represented higher ratings were given to the high fertility face compared to the low fertility face, while larger, negative scores indicate higher ratings were given to the low fertility face compared to the high fertility face. For each judgement, we conducted a linear mixed effect model, where the outcome was the difference score, while the predictors were the choices in the 3AFC task. Age and sex were included as covariates. These models assess whether participants who chose high fertility cues as the most attractive/feminine via 3AFC also rated high fertility images as more attractive on the 7-point scale.

Across the attractiveness models, we found only one significant, positive association between tasks when participants made judgements of the “textbook” fertility cues. For the femininity models, only judgements of the E/P ratio stimuli were significantly associated between the two tasks. There were no other significant associations between responses on the rating task and the 3AFC for any other judgements (Table 3).

**Table 3:** Associations between choice on the 3AFC task and ratings task for each of the separate models (statistically significant differences in bold).

	Estimate (SE)	*P*
**Attractiveness**		
Conception probability in all cycles	.02 (.08)	.840
**Conception probability in “textbook” cycles**	**.18 (.08)**	**.031**
E	− .05 (.08)	.516
P	− .08 (.08)	.302
E/P	− .06 (.08)	.555
**Femininity**		
Conception probability in all cycles	.23 (.09)	.086
Conception probability in “textbook” cycles	.04 (.09)	.660
E	.16 (.12)	.286
P	.09 (.09)	.287
**E/P**	**.17 (.09)**	**.046**

#### Are associations between fertility cues and judgements of attractiveness/femininity moderated by individual differences?

For each judgement (femininity and attractiveness for each of the five fertility cues) and task (3AFC and rating), we conducted two separate models, which resulted in 20 models in total. This included: (1) individual differences model, where age, sex, self-rated attractiveness, self-rated health, and self-rated financial difficulties were included as predictors, and (2) SOI model, where age, sex and sociosexual orientation were included as predictors. SOI was included in a separate model to maximise power as this variable had substantially more missing data compared to the other individual differences. For the 3AFC models, we report the main effect of individual difference (i.e., do individual differences influence choice for high, medium and low fertility cue). For the rating models, we report the interaction between level of fertility cue (high vs. low) and individual difference (i.e., do individual differences in age moderate the association between fertility cue and attractiveness/femininity rating).

For the 3AFC, self-rated attractiveness, self-rated health, financial difficulties, and SOI were not associated with attractiveness or femininity judgements for any of the fertility cues. Similarly, self-rated attractiveness, self-rated health, financial difficulties, and SOI did not moderate the association between fertility levels and either of the ratings. We note that while there were some significant age and sex associations between fertility cues and judgements of attractiveness/femininity, these were not consistent between models nor task types (for full results see ESM2).

#### Do population factors moderate the association between attractiveness/femininity judgements and cues to fertility?

To assess whether country level factors moderate the association between attractiveness/femininity judgements and cues to fertility, we first computed an analysis of health/development factor and an inequality factor based on country statistics via an Independent Factor Analysis (IFA), following the procedure in Marcinkowska et al. (2019). The only exception to this was that we included data for all available countries, not just those included in this dataset. The IFA originally included 121 countries to develop the factor scores; from this, the factor scores for the 12 countries included in this sample were taken. Based on results from previous cross-cultural studies the IFA included 11country demographic statistics, including the Human Development Index, life expectancy, years lost to disease, fertility rate, the Gender Inequality Index (GII), urbanisation, historical pathogen prevalence, mortality rate, homicide rate, Gini coefficient, and GDP. The IFA resulted in two factors, a health/development factor, and an inequality factor, which were included as predictors in the following models.

The only significant association between country factors and judgments of fertility was in the 3AFC task, and it was a significant association between the inequality factor and preference for high E, such that higher inequality was associated with reduced preference for E. There were no other significant associations between country factors and facial judgements.

## Discussion

In the current study, using a cross-cultural sample we tested the perception of women’s putative facial cues to fertility during three stages of menstrual cycle varying in conception probability. Specifically, we investigated whether perceptions of attractiveness and femininity in women’s faces are a function of menstrual cycle stage (follicular, peri-ovulatory or mid-luteal), measured by LH and E levels. Furthermore, due to the contradictory findings in the previous research regarding the association of sex hormones and women’s facial perception e.g.^11,38^, the secondary aim of the current study was to shed light on the controversy by employing both a 3AFC task as well as a rating task (i.e. Likert scale). We also tested for the potential influence of individual and environmental factors on facial preferences.

### Do faces convey signals to current fertility?

Our results suggest that peri-ovulatory phase prototypes in the forced choice task (but not in the rating task), as well as high E prototypes in the rating task (but not in forced choice task) were chosen as more attractive and feminine, providing results in accordance with the prediction that women’s faces are considered more attractive and feminine around ovulation, when conception probability is high. However, the differences in judgements between facial images from 3 phases of the cycle were significant only when narrowing the analyses to women who reported positive LH test results and experienced an E drop afterwards. Further work is needed to establish if these differences in results across analyses is meaningful or if the significant results observed are simply false positives due to the multiple tests carried out. That the effects that were statistically significant had extremely small effect sizes is consistent with a ‘false positive’ explanation^39^.

Additionally, the discrepancy between the results of forced choice and rating tasks highlights the task dependency of facial judgements. For example, while some previous research using force choice and ranking tasks failed to find any association between conception risk and attractiveness e.g.^8,9^, others using rating task have found this association e.g.^11^. Accordingly, here we provide empirical evidence supporting the argument that the judgements might differ according to study task^25^.

Aside from methodological issues related to task type, another source of results discrepancy can stem from measurements of facial characteristics. In recent study using computational approach facial shape was showed to be stable throughout the menstrual cycle^40^. Whatever the fluctuations in facial representation of fertility, they must be attenuated by the lack of specific trend in facial shape changes. Although aforementioned study focused on shape solely, it is possible, that the differences in perception observed in this study stem from changes in facial pigmentation or texture but see ^41,42^. Therefore, neither colour nor facial shape may be an honest que to current fertility. This evidence seems to be against the idea that selection favours males that are able to detect the female fertile period^6^ and is more in accordance with the hypothesis that the female mating period is mostly concealed, possibly to secure monogamy and paternal care^43^. An alternative hypothesis is that facial shape and pigmentation may reveal health^44–46^, but see^47^, rather than fertility. These topics require further research.

Contrary to our prediction, low E/P prototypes were considered more attractive and feminine (however only in 3AFC and not in rating task). Low E/P ratios are characteristic for luteal phase, where conception probability is low. If facial attractiveness was closely related to the underlining fertility, we should observe and opposite direction of this trend. It is possible, that due to the within-individual variation in daily hormonal levels, a 3 point measurements approach is burdened with excessive amount of noise caused by the daily fluctuations of both sex hormones. A study based on a higher number of daily measurements and photographs could add more information on the facial representation of the hormonal underpinnings.

### Do individual differences moderate facial cognition?

We investigated the effects of individual differences (i.e., sex, age, self-rated attractiveness, self-rated health, self-rated financial difficulties, and SOI) on preferences for facial fertility cues, as well as the potential moderating effects of such variables on the association between facial fertility cues and perceptions of attractiveness and femininity. Most results did not show significant effects for the association between individual differences and judgements of attractiveness or femininity other than sex differences (expected to be observed as analyses was based on solely heterosexual participants, and solely on female facial images).

The findings on relation between age and facial preferences were mixed. While there was a negative effect of age on fertility preferences based on the entire sample, there was a positive effect of age on high E/P (i.e. a faces of high E/P ratio typical for increased fertility were chosen more often as attractive by older men than by younger men). While some previous studies have found that the age of the subject is an important factor in mate preferences^48,49^, others have found that the ratters’ age did not matter in attractiveness and fertility ratings^50^. A study designed entirely to test the effect of age on facial cognition could help to understand possible moderators, such as self-rated attractiveness, socio-economic status or family composition affecting the intricate relationship.

### Cross-cultural variation in fertility perception

The analysis for the country level factors showed a significant association between the inequality factor and preference for high E, indicating participants from the countries with higher inequality preferred low E prototype face. This association supports the previous research showing preference for less feminine female faces as the result of harsher environments^17,51^, but see^52^. Such a mechanism could serve to direct attention to putative partners who are better equipped to compete for resources (as less feminine women were described^53^. Alternatively, as suggested by Marcinkowska et al. (2014) harshness of living conditions can decrease preference for femininity via lowered testosterone levels. Harshness of living conditions and prevalence of pathogens is negatively related to testosterone^54^ and testosterone in turn correlates with preferences for femininity^55^. Lowered attraction to faces with low E (and low femininity what follows) could be a by-product of lowered levels of sex hormones in men. However, this study provides only indirect evidence for the idea, as levels of sex hormones of ratters were not measured.

It should be noted that models used in the current paper were based on observations from 12 countries, and lack of enough data points at country level might have resulted in failure in capturing a more nuanced variance. For example, while previous cross-cultural research has reported the existence of the relationship between sociosexuality and mate preferences^14,56^, we were not able to find such association. We therefore suggest further research is required that includes participants from a larger number of countries.

## Conclusions and future directions

This cross-cultural study employing two methodological approaches provided somewhat limited support for the hormonal underpinnings of facial femininity and attractiveness fluctuations throughout cycle. Although some results followed the previously suggested positive relationship between conception probability and attractiveness, there was by far no consistency between task types and between models. Furthermore, some results, i.e. E/P ratio relation with attractiveness was not only non-significant, but was actually significant in the opposite direction than predicted.

The exploratory nature of this research warrants a need for verifying currently found results via high powered replication, especially as the results found are inconsistent depending on the task type. For all analyses, we maintained an alpha of *p* < .05 as we wished to prioritise identifying potential effects that could be explored in future, more targeted research. We note that, given the number of models, some significant effects would not survive corrections for multiple comparisons. We also note that the effect size of significant effects are small, and should be interpreted very cautiously^57^. For example, it is unlikely that such small changes in women’s facial appearance, even if robust, would be detectable during real world social interactions.

As showed in this study, the fluctuations in sex hormones do not provide sufficient explanation for previously found cyclic changes in facial perception. Because the up-to-date state of art presents conflicting results (in line with the pattern of results obtained in this study) we believe that more studies are needed before establishing scientifically well-grounded relation between putative facial cues to women’s’ fertility and femininity and attractiveness.

## Supplementary Information

Supplementary Information 1.

Supplementary Information 2.

## Data Availability

The datasets generated during and analysed during the current study are available on the OSF page of the project which can be accessed on the link: https://osf.io/g2ve5/.
