# Publisher Correction: An exploratory, cross-cultural study on perception of putative cyclical changes in facial fertility cues

**DOI:** 10.1038/s41598-021-97722-5

**Published:** 2021-09-06

**Authors:** Urszula M. Marcinkowska, Benedict C. Jones, Huaijan Cai, Jorge Contreras‑Garduno, Ike E. Onyishi, Charles T. Orjiakor, Keshav Prasai, Farid Pazhoohi, Hirokazu Taniguchi, Anthony J. Lee

**Affiliations:** 1 Department of Health Sciences, Jagiellonian University Medical College, Cracow, Poland; 2 School of Psychology, University of Strathclyde, Glasgow, Scotland, UK; 3 Institute of Psychology, Chinese Academy of Sciences, Beijing, People’s Republic of China; 4 ENES Campus Morelia, National Autonomous University of Mexico, Morelia, Mexico; 5 Department of Psychology, University of Nigeria, Nsukka, Nigeria; 6 Uniglobe H.S.S/College, Kathmandu, Nepal; 7 Department of Psychology, University of British Columbia, Vancouver, Canada; 8 Shimonoseki City University, Shimonoseki, Japan; 9 Division of Psychology, University of Stirling, Stirling, Scotland, UK

Correction to: *Scientific Reports* 10.1038/s41598-021-96454-w, published online 19 August 2021

The Funding section in the original version of this Article was omitted. The Funding section now reads:

“This work was funded by the Polish National Science Center (grant number 2014/12/S/NZ8/00722), and the Polish-U.S. Fulbright Commission (grant number PL/2018/42/SR).”

The original Article has been corrected.

