# Supplementary material for: An exploratory, cross-cultural study on perception of putative cyclical changes in facial fertility cues
Source: Sci Rep. 2021 Aug 19;11:16911. doi: 10.1038/s41598-021-96454-w (PMC8377019; doi:10.1038/s41598-021-96454-w)
Supplement: Supplementary file 1 — Supplementary Information 1. [file 41598_2021_96454_MOESM1_ESM.pdf]

An exploratory, cross-cultural study on perception of putative cyclical changes in facial fertility cues. (Data Prep)

Contents

Load Packages and Custom Functions 1

Load Data 2
- Country Codes for Joining Data . . . 2
- UK/Mexico Data . . . 2
- China Data . . . 3
- Cross-Cultural Data . . . 4
- Nigeria Data . . . 5

Prepare Dataset for Analysis 6
- Join Datasets . . . 6
- Filter Participants . . . 6
- Country-level Statistics . . . 6
- Participant-Level Descriptive Statistics . . . 7
- Participant Distribution by Country . . . 8
- 3 Alternative Forced Choice Data . . . 8
- Rating Data . . . 8

Save Workspace 9

Load Packages and Custom Functions

```
library(tidyverse)
library(lubridate)
library(rworldmap)
library(lme4)
library(lmerTest)
library(ordinal)

coords2country <- function(.data){
    points <- select(.data,LocationLongitude,LocationLatitude)
    points$LocationLongitude <- points$LocationLongitude %>% as.numeric()
    points$LocationLatitude <- points$LocationLatitude %>% as.numeric()
    countriesSP <- getMap(resolution = 'low')
    pointsSP <- SpatialPoints(points,proj4string=CRS(proj4string(countriesSP)))
    indices <- sp::over(pointsSP,countriesSP)
    .data$country_name <- as.character(indices$ADMIN)
    return(.data)
}

recode.country <- function(x){
  out <- recode(x,"Iran, Islamic Republic of..." = "Iran",
```

```
                    "United Kingdom of Great Britain and Northern Ireland" = "United Kingdom")
}

z <- function(x,remove.outliers = FALSE,winsorise = FALSE){
    out <- (x - mean(x,na.rm = TRUE))/sd(x,na.rm = TRUE)
    if (remove.outliers == TRUE){
      out <- ifelse(out >3,NA,ifelse(out < -3,NA,out))
    }
    if (winsorise == TRUE){
      out <- ifelse(out > 3,3,ifelse(out < -3,-3,out))
    }
    return(out)
}

correlations <- function(.data,variables,with=variables){
    .data <- as.data.frame(.data)
    vnames <- vector(mode="character",length=4*NROW(variables))
    for (v in 1:NROW(variables)){
        vnames[((v-1)*4)+2] <- variables[v]
    }
    output <- data.frame("variable"=vnames)
    for (w in 1:NROW(with)){
        print <- vector(mode="character",length=4*NROW(variables))
        for (v in 1:NROW(variables)){
            cor <- cor.test(.data[,variables[v]],.data[,with[w]])
            print[((v-1)*4)+2] <- paste("r = ",round(cor$estimate,3),sep="")
            print[((v-1)*4)+3] <- paste("p = ",round(cor$p.value,3),sep="")
            print[((v-1)*4)+4] <- paste("N = ",round(cor$parameter,3)+2,sep="")
        }
        output <- cbind(output,print)
    }
    colnames(output) <- c("variables",with)
    output
}
```

Load Data

Country Codes for Joining Data

```
country.codes <- read.csv("Country Codes.txt",stringsAsFactors = FALSE,header = FALSE)

colnames(country.codes) <- c("country","country_name")
```

UK/Mexico Data

```
data_UK <- read.csv("joined_dataUKMexico.csv",stringsAsFactors = FALSE) %>%
    left_join(country.codes,by = "country") %>%
    select(endtime,user_id,sex,DOB,country_name,orientation,relationship,rel_satisfaction,kids,financia
    mutate(soi1 = as.numeric(NA),soi2 = as.numeric(NA),soi3 = as.numeric(NA),soi4 = as.numeric(NA),soi5
    mutate( endtime = ymd_hms(endtime),
            DOB = date_decimal(DOB),
            data.set = "UK",
```

```
            user_id = as.character(user_id))
```

China Data

```
data_CH <- read.csv("Men's preferences Crossculturally Chinese+NepaliRAW.csv",stringsAsFactors = FALSE)
    filter(!is.na(Status)) %>%
    filter(!is.na(LocationLatitude) & !is.na(LocationLongitude) & LocationLatitude != "" & LocationLong
    #filter(Progress >= 99) %>%
    coords2country() %>%
    mutate(
            endtime = dmy_hm(EndDate),
            user_id = ResponseId,
            sex = recode(Q2.2    ,"2" = "0",
                                  "1" = "1",
                                  "3" = "2") %>% as.numeric(),
            DOB = date_decimal(2001 - Q2.3),
            orientation = Q2.7,
            relationship = Q2.8,
            rel_satisfaction = Q2.9_1,
            kids = Q2.10,
            financial_dificulties = Q2.11,
            sra = Q2.12,
            srh = Q2.13,
            pregnant = Q2.14,
            breastfeeding = Q2.15,
            menopause = Q2.16,
            cycle_length = Q2.17,
            days_since = Q2.18,
            regular = Q2.19,
            HC = Q2.20,
            all_att_3afc = Q3.2,
            strict_textbook_att_3afc = Q3.3,
            E2_att_3afc = Q3.4,
            P_att_3afc = Q3.5,
            EtoP_att_3afc = Q3.6,
            all_I_att_rating = Q3.7,
            all_II_att_rating = Q3.8,
            all_III_att_rating = Q3.9,
            E2_low_att_rating = Q3.10,
            E2_high_att_rating = Q3.11,
            P_low_att_rating = Q3.12,
            P_high_att_rating = Q3.13,
            EtoP_low_att_rating = Q3.14,
            EtoP_high_att_rating = Q3.15,
            strict_textbook_I_att_rating = Q3.16,
            strict_textbook_II_att_rating = Q3.17,
            strict_textbook_III_att_rating = Q3.18,
            all_fem_3afc = Q4.2,
            strict_textbook_fem_3afc = Q4.3,
            E2_fem_3afc = Q4.4,
            P_fem_3afc = Q4.5,
            EtoP_fem_3afc = Q4.6,
            all_I_fem_rating = Q4.7,
```

```
            all_II_fem_rating = Q4.8,
            all_III_fem_rating = Q4.9,
            E2_low_fem_rating = Q4.10,
            E2_high_fem_rating = Q4.11,
            P_low_fem_rating = Q4.12,
            P_high_fem_rating = Q4.13,
            EtoP_low_fem_rating = Q4.14,
            EtoP_high_fem_rating = Q4.15,
            strict_textbook_I_fem_rating = Q4.16,
            strict_textbook_II_fem_rating = Q4.17,
            strict_textbook_III_fem_rating = Q4.18,
            soi1 = Q5.2,
            soi2 = Q5.3,
            soi3 = Q5.4,
            soi4 = Q5.5,
            soi5 = Q5.6,
            soi6 = Q5.7,
            soi7 = Q5.8,
            soi8 = Q5.9,
            soi9 = Q5.10,
            data.set = "CH") %>%
    select(endtime,user_id,sex,DOB,country_name,orientation,relationship,rel_satisfaction,kids,financia
```

Cross-Cultural Data

```
data_RAW <- read.csv("Men's preferences CrosscultQualtrics011119Numeric.csv",stringsAsFactors = FALSE) 
    #filter(row_number() > 2) %>%
    filter(!is.na(LocationLatitude) & !is.na(LocationLongitude) & LocationLatitude != "" & LocationLong
    #filter(Progress >= 99) %>%
    coords2country() %>%
    mutate(
            #country_name = "test",
            endtime = ymd_hms(EndDate),
            user_id = ResponseId,
            sex = recode(Q2.2   ,"2" = "0",
                                 "1" = "1",
                                 "3" = "2") %>% as.numeric(),
            DOB = date_decimal(2001 - Q2.3),
            orientation = Q2.7,
            relationship = Q2.8,
            rel_satisfaction = Q2.9_1,
            kids = Q2.10,
            financial_dificulties = Q2.11,
            sra = Q2.12,
            srh = Q2.13,
            pregnant = Q2.14,
            breastfeeding = Q2.15,
            menopause = Q2.16,
            cycle_length = Q2.17,
            days_since = Q2.18,
            regular = Q2.19,
            HC = Q2.20,
            all_att_3afc = Q3.2,
```

```
            strict_textbook_att_3afc = Q3.3,
            E2_att_3afc = Q3.4,
            P_att_3afc = Q3.5,
            EtoP_att_3afc = Q3.6,
            all_I_att_rating = Q3.7,
            all_II_att_rating = Q3.8,
            all_III_att_rating = Q3.9,
            E2_low_att_rating = Q3.10,
            E2_high_att_rating = Q3.11,
            P_low_att_rating = Q3.12,
            P_high_att_rating = Q3.13,
            EtoP_low_att_rating = Q3.14,
            EtoP_high_att_rating = Q3.15,
            strict_textbook_I_att_rating = Q3.16,
            strict_textbook_II_att_rating = Q3.17,
            strict_textbook_III_att_rating = Q3.18,
            all_fem_3afc = Q4.2,
            strict_textbook_fem_3afc = Q4.3,
            E2_fem_3afc = Q4.4,
            P_fem_3afc = Q4.5,
            EtoP_fem_3afc = Q4.6,
            all_I_fem_rating = Q4.7,
            all_II_fem_rating = Q4.8,
            all_III_fem_rating = Q4.9,
            E2_low_fem_rating = Q4.10,
            E2_high_fem_rating = Q4.11,
            P_low_fem_rating = Q4.12,
            P_high_fem_rating = Q4.13,
            EtoP_low_fem_rating = Q4.14,
            EtoP_high_fem_rating = Q4.15,
            strict_textbook_I_fem_rating = Q4.16,
            strict_textbook_II_fem_rating = Q4.17,
            strict_textbook_III_fem_rating = Q4.18,
            soi1 = Q5.2,
            soi2 = Q5.3,
            soi3 = Q5.4,
            soi4 = Q5.5,
            soi5 = Q5.6,
            soi6 = Q5.7,
            soi7 = Q5.8,
            soi8 = Q5.9,
            soi9 = Q5.10,
            data.set = "RAW") %>%
    select(endtime,user_id,sex,DOB,country_name,orientation,relationship,rel_satisfaction,kids,financia
```

Nigeria Data

```
data_NI <- read.csv("Data Facial Preference Oct2018NIgeria.csv",stringsAsFactors = FALSE)

colnames(data_NI) <- c("user_id","sex","DOB","ethnicity","country_name","orientation","relationship","r

data_NI$data.set <- "NI"
data_NI$user_id <- 1:NROW(data_NI)
```

```
data_NI <- data_NI %>%
    #filter(country_name == "Nigeria") %>%
    mutate(
    DOB = date_decimal(DOB),
    user_id = paste("NI",user_id,sep = "_"),
    sex = recode(sex,"1" = "1","2" = "0","3" = "2") %>% as.numeric(),
    HC = recode(HC,"2" = "2","No" = "1") %>% as.numeric(),
    endtime = as.POSIXct(NA))
```

Prepare Dataset for Analysis

Join Datasets

```
full.data <- bind_rows(data_UK,data_CH) %>%
    bind_rows(data_RAW) %>%
    bind_rows(data_NI)
write.csv(full.data,"full.data.csv",row.names = FALSE)
```

Filter Participants

```
analysis.data <- full.data %>%
    filter(orientation <= 3) %>%
    filter(sex != 2) %>%
    group_by(country_name) %>%
    filter(n() >= 10) %>%
    ungroup() %>%
  mutate(sex = as.numeric(recode(sex,"0" = "-.5","1" = ".5")),
        age = year(ymd_hms(endtime)) - year(ymd(DOB)),
        soi = soi1 + soi2 + soi3 + soi4 + (soi5*-1 + 10) + soi6 + soi7 + soi8 + soi9,
        z.age = z(age,winsorise = TRUE),
        z.soi = z(soi, winsorise = TRUE),
        z.sra = z(sra, winsorise = TRUE),
        z.srh = z(srh, winsorise = TRUE),
        z.financial_difficulties = z(financial_dificulties, winsorise = TRUE),
        z.kids = z(kids))
```

Country-level Statistics

```
country.data <- read.csv("Updated Country.Data.csv",stringsAsFactors = FALSE) %>%
  mutate(Country = recode.country(Country)) %>%
  filter(Country %in% analysis.data$country_name) %>%
  mutate(health.fa = z(health.fa,winsorise = TRUE),
        inequality.fa = z(inequality.fa,winsorise = TRUE)) %>%
  select(Country,region,health.fa,inequality.fa)

analysis.data <- mutate(analysis.data,Country = recode.country(country_name)) %>%
  left_join(country.data,by = "Country")
```

Participant-Level Descriptive Statistics

```
correlations(analysis.data,variables = c("sex","age","soi","sra","srh","financial_dificulties","kids"))
```

```
##                   variables        sex        age       soi        sra        srh
## 1
## 2                       sex      r = 1  r = 0.024 r = 0.135 r = -0.038 r = -0.038
## 3                                p = 0    p = 0.4     p = 0  p = 0.161  p = 0.162
## 4                             N = 1371   N = 1188   N = 728   N = 1368   N = 1365
## 5
## 6                       age  r = 0.024      r = 1 r = 0.107  r = 0.005  r = 0.089
## 7                              p = 0.4      p = 0 p = 0.012  p = 0.858  p = 0.002
## 8                             N = 1188   N = 1188   N = 557   N = 1186   N = 1185
## 9
## 10                      soi  r = 0.135  r = 0.107     r = 1  r = 0.005   r = -0.03
## 11                               p = 0  p = 0.012     p = 0  p = 0.901  p = 0.418
## 12                             N = 728    N = 557   N = 728    N = 725    N = 723
## 13
## 14                      sra r = -0.038  r = 0.005 r = 0.005      r = 1  r = 0.473
## 15                           p = 0.161  p = 0.858 p = 0.901      p = 0      p = 0
## 16                            N = 1368   N = 1186   N = 725   N = 1368   N = 1364
## 17
## 18                      srh r = -0.038  r = 0.089 r = -0.03  r = 0.473      r = 1
## 19                           p = 0.162  p = 0.002 p = 0.418      p = 0      p = 0
## 20                            N = 1365   N = 1185   N = 723   N = 1364   N = 1365
## 21
## 22 financial_dificulties r = -0.009 r = -0.124 r = 0.031 r = -0.123 r = -0.133
## 23                           p = 0.741      p = 0 p = 0.417      p = 0      p = 0
## 24                            N = 1313   N = 1157   N = 688   N = 1312   N = 1311
## 25
## 26                     kids  r = 0.108  r = 0.358 r = 0.121 r = -0.048 r = -0.076
## 27                               p = 0      p = 0 p = 0.001  p = 0.075  p = 0.005
## 28                            N = 1358   N = 1178   N = 722   N = 1356   N = 1353
##    financial_dificulties       kids
## 1
## 2             r = -0.009  r = 0.108
## 3              p = 0.741      p = 0
## 4               N = 1313   N = 1358
## 5
## 6             r = -0.124  r = 0.358
## 7                  p = 0      p = 0
## 8               N = 1157   N = 1178
## 9
## 10             r = 0.031  r = 0.121
## 11             p = 0.417  p = 0.001
## 12               N = 688    N = 722
## 13
## 14            r = -0.123 r = -0.048
## 15                 p = 0  p = 0.075
## 16              N = 1312   N = 1356
## 17
## 18            r = -0.133 r = -0.076
## 19                 p = 0  p = 0.005
## 20              N = 1311   N = 1353
```

```
## 21
## 22                   r = 1 r = -0.048
## 23                   p = 0  p = 0.082
## 24                N = 1313   N = 1305
## 25
## 26             r = -0.048      r = 1
## 27              p = 0.082      p = 0
## 28               N = 1305   N = 1358
```

Participant Distribution by Country

```
analysis.data %>%
  group_by(Country) %>%
  summarise(N = n(),
            n_males = sum(sex == .5),
            n_females = sum(sex == -.5),
            mean_age = mean(age,na.rm = TRUE),
            sd_age = sd(age,na.rm = TRUE))
```

```
## # A tibble: 13 x 6
##    Country                      N n_males n_females mean_age sd_age
##    <chr>                    <int>   <int>     <int>    <dbl>  <dbl>
##  1 Australia                   11       7         4     35.9  6.47
##  2 China                      230     130       100     32.3  7.37
##  3 Germany                     10       3         7     32.7 11.0
##  4 Iran                        51      17        34     29.3 12.6
##  5 Japan                       82      23        59     33.9  0.553
##  6 Mexico                     670     433       237     27.3  9.11
##  7 Nepal                       40      35         5     29.4  7.77
##  8 Nigeria                    190      84       106     16.6  5.97
##  9 Poland                      18       9         9     41.6 11.2
## 10 Spain                       10       5         5     33.6  7.00
## 11 United Kingdom              17       6        11     33.2 12.5
## 12 United States of America    24      17         7     41.5 21.5
## 13 <NA>                        18       8        10     26.9 10.1
```

3 Alternative Forced Choice Data

```
afc.data <- analysis.data  %>%
    select(user_id,country_name,sex,age = z.age,soi = z.soi,sra = z.sra,srh = z.srh,financial_dificulti

    gather(key = "trial",value = "choice",all_att_3afc:EtoP_fem_3afc) %>%
    mutate(trial = gsub("strict_textbook","textbook",trial)) %>%
    separate(trial,into = c("Type","Judgement","Task"),sep = "_") %>%
    mutate(choice = recode(choice,`1` = -.5,`2` = 0,`3` = .5,`4` = NULL,.missing = NULL)) %>%
  filter(!is.na(choice))
```

Rating Data

```
rating.data <- analysis.data %>%
    select(user_id,country_name,sex,age = z.age,soi = z.soi,sra = z.sra,srh = z.srh,financial_dificulti
    gather(key = "trial",value = "rating",all_I_att_rating:strict_textbook_III_fem_rating) %>%
    mutate(trial = gsub("strict_textbook","textbook",trial)) %>%
```

```
    separate(trial,into = c("Type","level","Judgement","Task"),sep = "_") %>%
    #filter(Type == "E2" | Type == "EtoP" | Type == "P") %>%
    mutate(level =  ifelse(level == "high",.5,
                    ifelse(level == "low",-.5,
                    ifelse(level == "I",-.5,
                    ifelse(level == "II",0,.5))))) %>%
  mutate(rating = ifelse(rating == 65,NA,rating))
```

Save Workspace

```
save(list = c("analysis.data","afc.data","rating.data"),file = "workspace.Rdata")
```
