# Supplementary material for: An exploratory, cross-cultural study on perception of putative cyclical changes in facial fertility cues
Source: Sci Rep. 2021 Aug 19;11:16911. doi: 10.1038/s41598-021-96454-w (PMC8377019; doi:10.1038/s41598-021-96454-w)
Supplement: Supplementary file 2 — Supplementary Information 2. [file 41598_2021_96454_MOESM2_ESM.pdf]

An exploratory, cross-cultural study on perception of putative cyclical changes in facial fertility cues (Results).

Contents

Are judgements of attractiveness and femininity associated with putative fertility cues? **1**
3AFC . . . 1
Rating . . . 12

Are judgements of attractiveness and femininity for fertility cues consistent between tasks? **16**
All . . . 16
Textbook . . . 17
E2 . . . 18
P . . . 19
EtoP . . . 20

Are associations between fertility cues and judgements of attractiveness and femininity moderated by individual differences? **22**
3 Alternative Forced Choice Data . . . 22
Rating Data . . . 29

Do country factors moderate the association between attractiveness and femininity judgements and cues to fertility? **40**
All . . . 40
Textbook . . . 41
E2 . . . 43
P . . . 45
EtoP . . . 46

```
library(tidyverse)
library(lubridate)
library(rworldmap)
library(lme4)
library(lmerTest)
library(ordinal)
library(ggtern)
library(knitr)

load("workspace.Rdata")
```

Are judgements of attractiveness and femininity associated with putative fertility cues?

3AFC

```
simulate_afc <- function(data,iter = 10000){
  N <- data %>% NROW()
```

```
  results <- vector(mode = "numeric",length = iter)
  simprops <- matrix(nrow = iter,ncol = 3)
  for(i in 1:iter){
    simprops[i,] <- sample(x = c(-.5,0,.5),size = N,prob = c(1/3,1/3,1/3),replace = TRUE) %>%       tab[illegible]
    results[i] <- sum((simprops[i,] - 1/3)^2)
  }

  obsprop <- data$choice %>% table()/N

  simprops <- as.data.frame(rbind(simprops,obsprop)) %>%
    mutate(data = "Simulated Data")
  colnames(simprops) <- c("Low","Neutral","High","data")
  simprops$data[NROW(simprops)] <- "Observed Data"

  PS <- ((data$choice %>% table()/N)*100) %>% round(2)
  names(PS) <- c("Low","Neutral","High")

  obs <- sum(((data$choice %>% table()/N) - 1/3)^2)
  pValue <- sum(results >= obs)/length(results) %>% round(3)

  title <- paste("p-value = ",pValue,sep = "")

  plot <- ggtern(data = simprops,aes(x = Low,y = Neutral,z = High,colour = data)) +
    geom_point() +
    ggtitle(title) +
    theme_zoom_center(.5) +
    #Tlab(paste("Neutral (",PS[2],"%)",sep = "")) +
    #Llab(paste("Low (",PS[1],"%)",sep = "")) +
    #Rlab(paste("High (",PS[3],"%)",sep = "")) +
    theme(legend.position = "bottom")

  return(list(plot,PS))
}

short <- function(s,title){
  if(class(s) == "summary.clmm"){
    x <- s$coefficients
    colnames(x)[4] <- "p value"
  } else {
    x <- s$coefficients
    colnames(x)[5] <- "p value"
  }

  out <- round(x,3) %>% kable(caption = title)
  return(out)
}

set.seed(53)
```

All

```
simulate_afc(filter(afc.data, Type == "all",Judgement == "att"))
```

```
## [[1]]
```

p−value = 0.8468

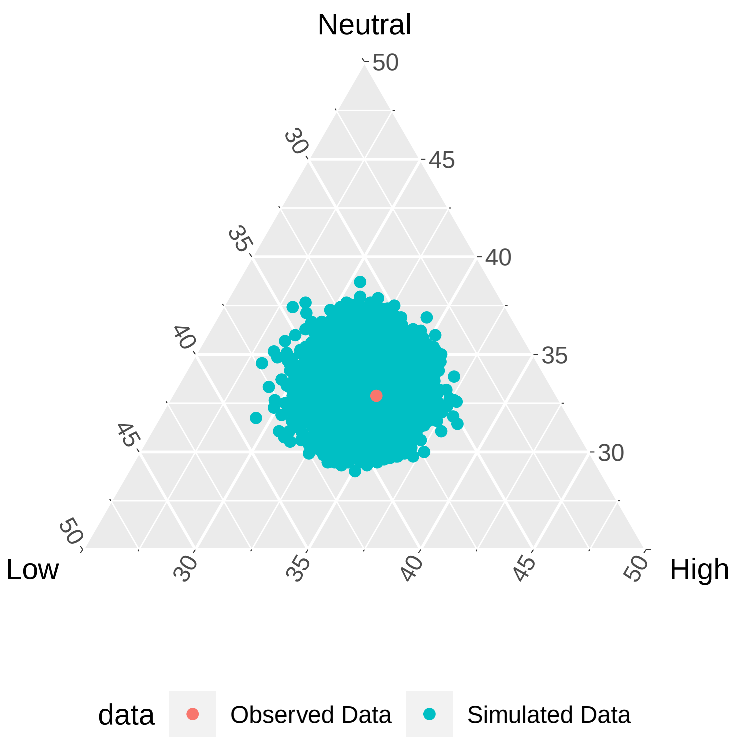


```
##
## [[2]]
##      Low Neutral    High
##    33.03   32.88   34.09
```

```
simulate_afc(filter(afc.data, Type == "all",Judgement == "fem"))
```

```
## [[1]]
```

p−value = 0.5753

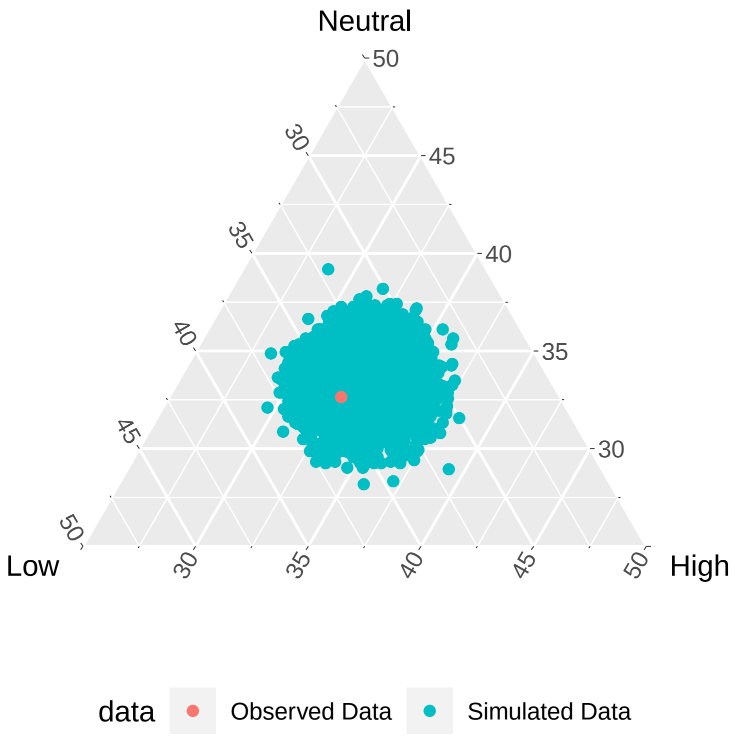


```
##
## [[2]]
##      Low Neutral    High
##   34.72   32.64   32.64
```

Textbook

```
simulate_afc(filter(afc.data, Type == "textbook",Judgement == "att"))
```

```
## [[1]]
```

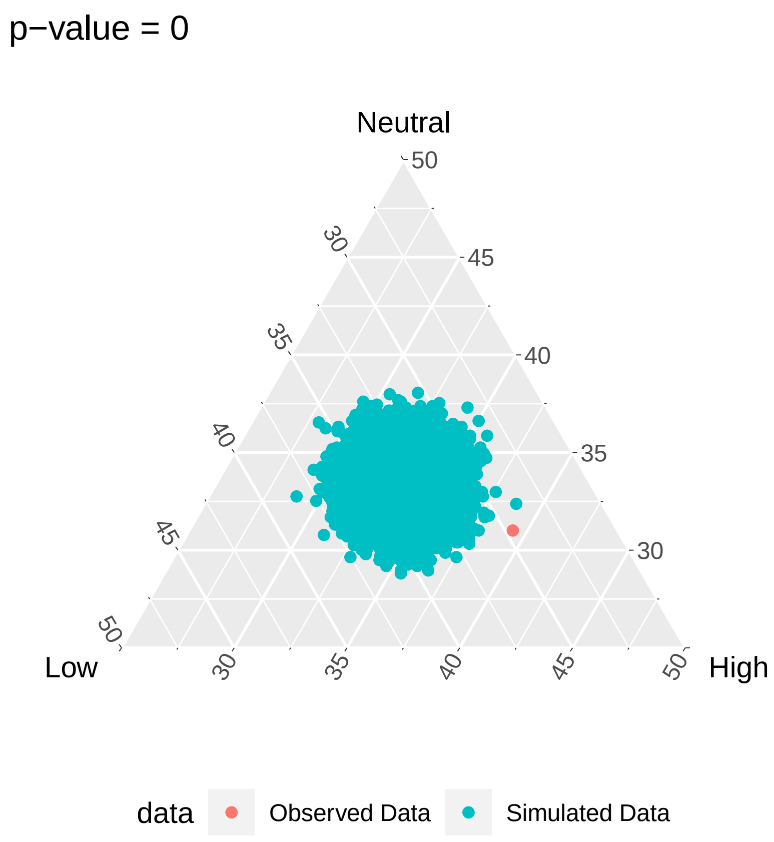


```
##
## [[2]]
##      Low Neutral    High
##   29.64   31.01   39.35
```

```
simulate_afc(filter(afc.data, Type == "textbook",Judgement == "fem"))
```

```
## [[1]]
```

p−value = 0.0472

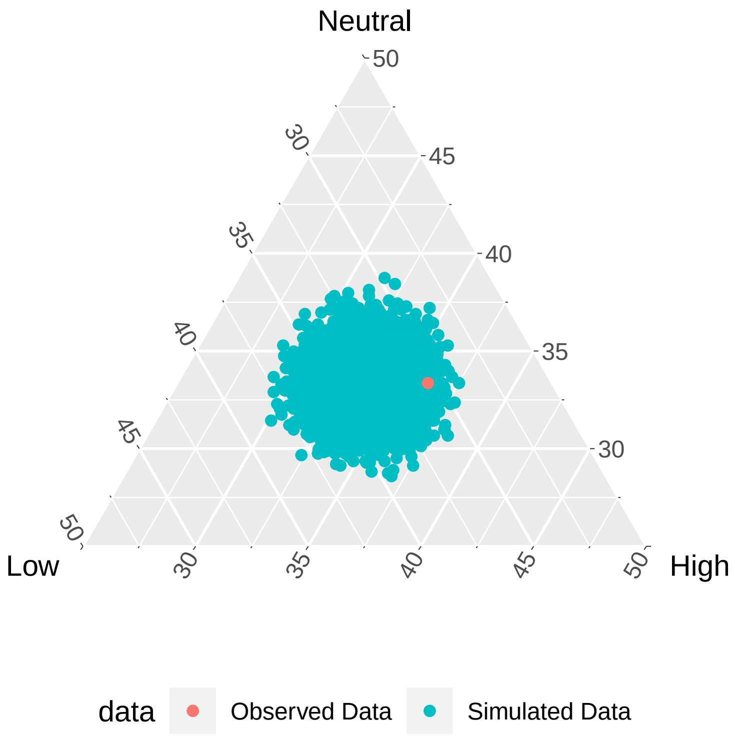


```
##
## [[2]]
##      Low Neutral    High
##   30.51   33.36   36.13
```

E2

```
simulate_afc(filter(afc.data, Type == "E2",Judgement == "att"))
```

```
## [[1]]
```

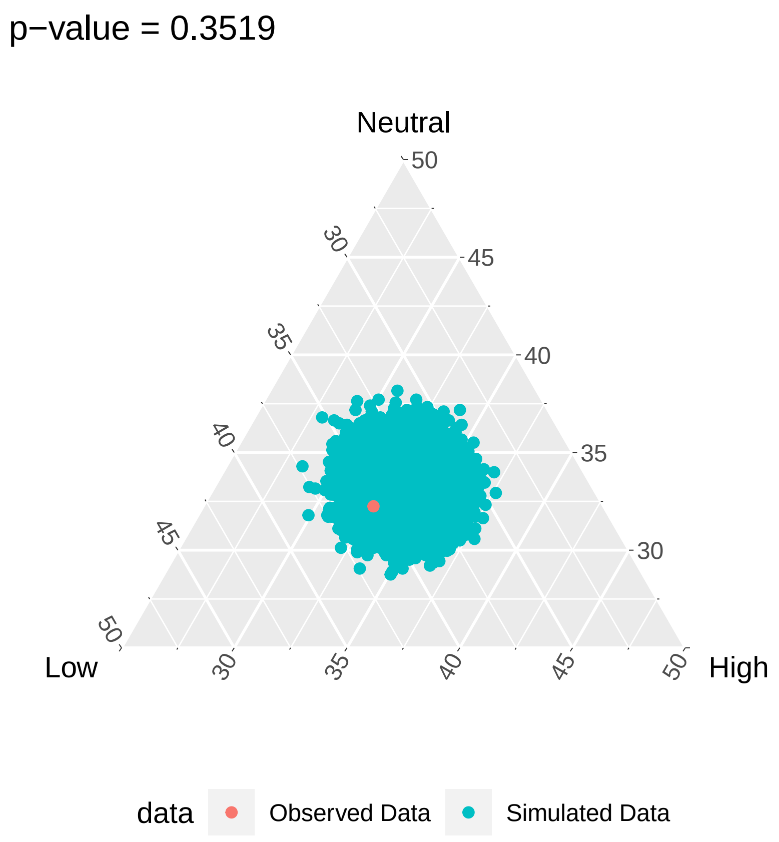


```
##
## [[2]]
##      Low Neutral    High
##    35.20   32.25   32.55
```

```
simulate_afc(filter(afc.data, Type == "E2",Judgement == "fem"))
```

```
## [[1]]
```

p−value = 0.3542

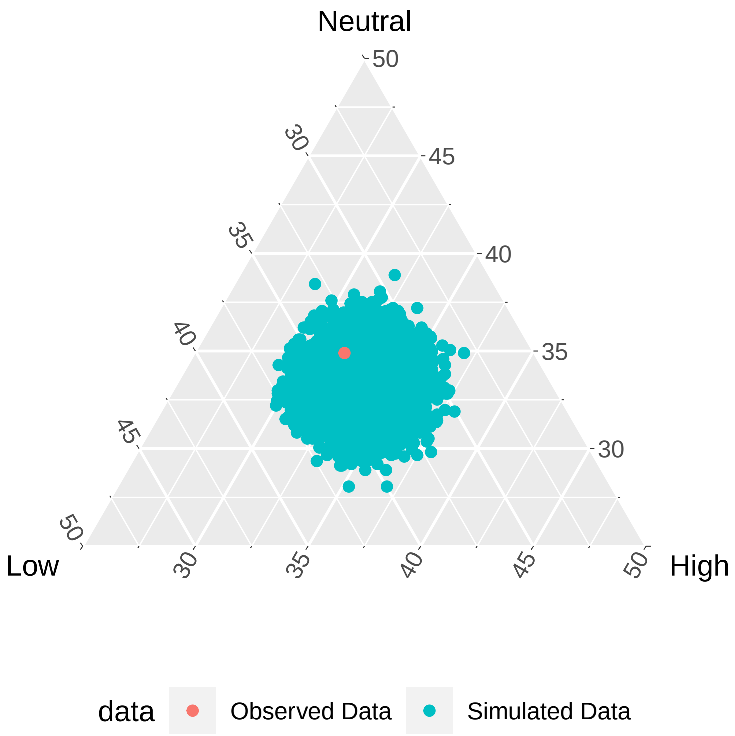


```
##
## [[2]]
##      Low Neutral    High
##    33.44   34.90   31.67
```

P

```
simulate_afc(filter(afc.data, Type == "P",Judgement == "att"))
```

```
## [[1]]
```

p−value = 0.2961

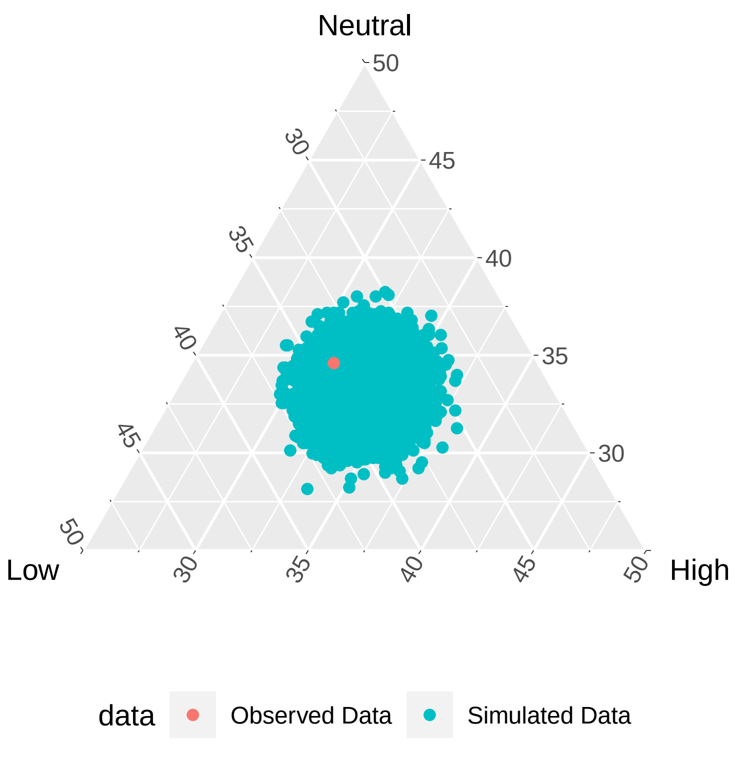


```
##
## [[2]]
##      Low Neutral    High
##    34.07   34.60   31.34
```

```
simulate_afc(filter(afc.data, Type == "P",Judgement == "fem"))
```

```
## [[1]]
```

p−value = 0.5331

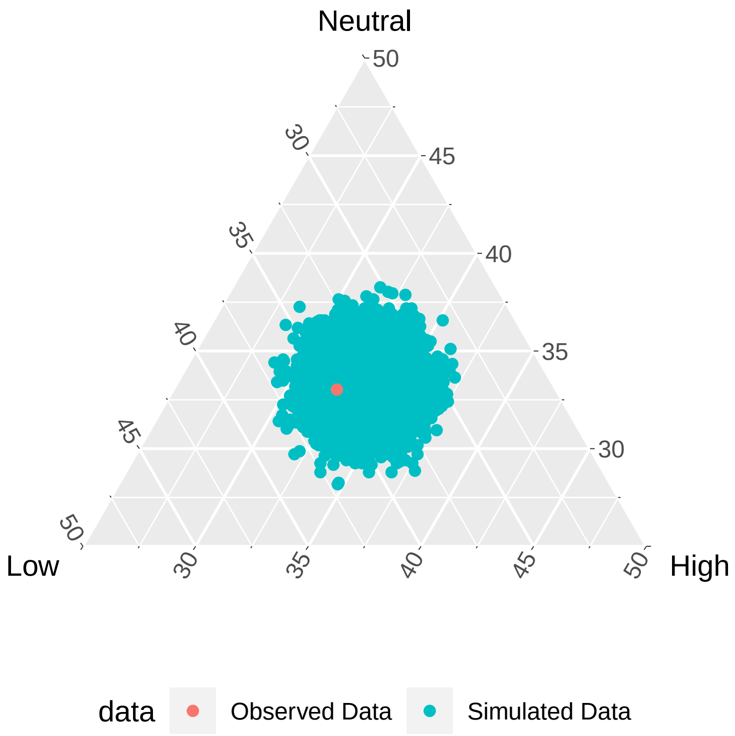


```
##
## [[2]]
##      Low Neutral    High
##    34.72   33.03   32.26
```

EtoP

```
simulate_afc(filter(afc.data, Type == "EtoP",Judgement == "att"))
```

```
## [[1]]
```

p−value = 0.0262

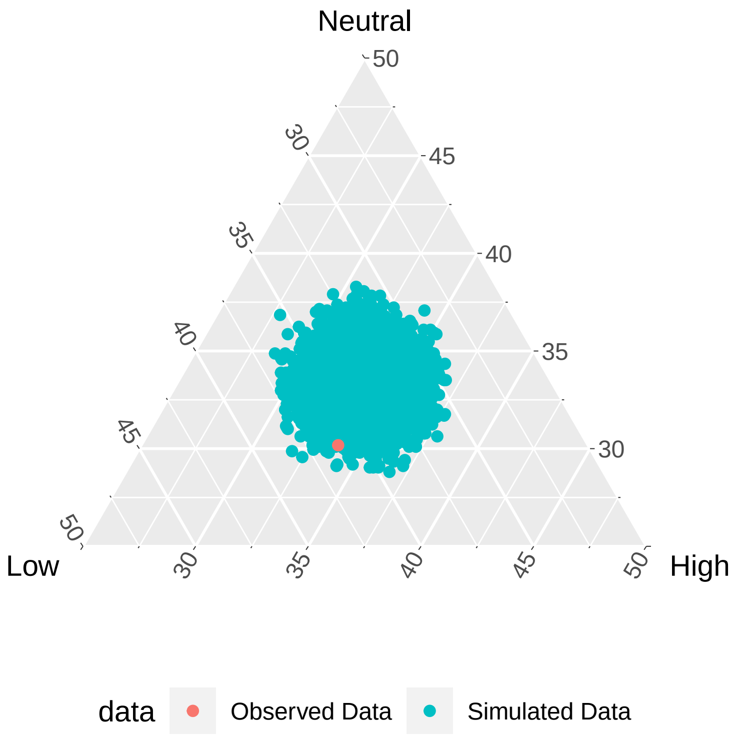


```
##
## [[2]]
##      Low Neutral    High
##   36.09   30.17   33.74
```

```
simulate_afc(filter(afc.data, Type == "EtoP",Judgement == "fem"))
```

```
## [[1]]
```

p−value = 0.0058

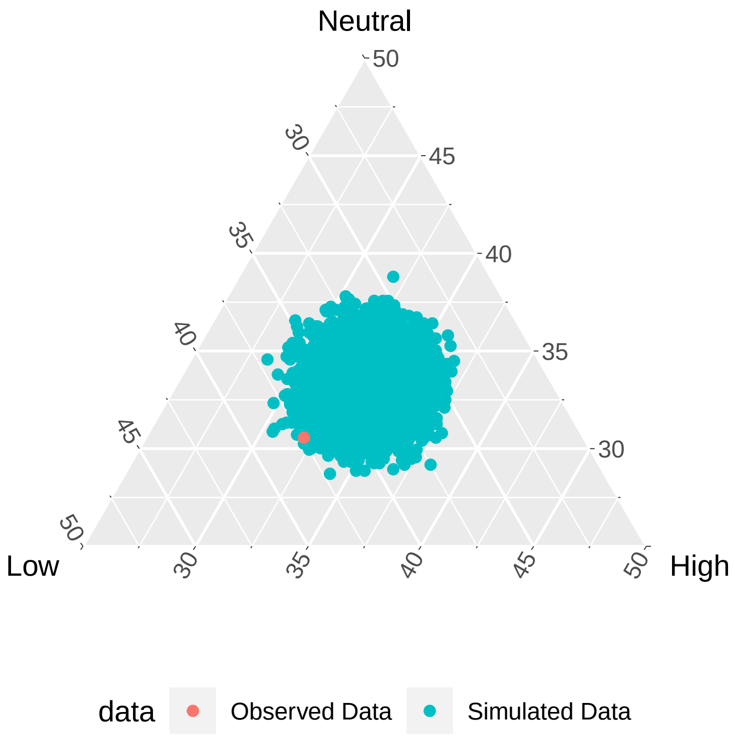


```
##
## [[2]]
##      Low Neutral    High
##    37.41   30.56   32.02
```

Rating

All

```
model_a1 <- lmer(rating ~ age + sex + level +
                  (1 + age + sex + level || country_name) +
                  (1 + level || user_id),data = filter(rating.data,Type == "all",Judgement == "att"))
```

```
## boundary (singular) fit: see ?isSingular
```

```
summary(model_a1) %>% short(title = "Attractiveness Ratings - All Fertility")
```

```
## Warning in if (class(s) == "summary.clmm") {: the condition has length > 1 and
## only the first element will be used
```

Table 1: Attractiveness Ratings - All Fertility

	Estimate	Std. Error	df	t value	p value
(Intercept)	4.309	0.113	9.635	38.156	0.000
age	0.183	0.046	0.656	3.983	0.250
sex	-0.061	0.071	918.877	-0.860	0.390
level	-0.040	0.035	1067.001	-1.163	0.245

```
model_a1 <- lmer(rating ~ age + sex + level +
                   (1 + age + sex + level || country_name) +
                   (1 + level || user_id),data = filter(rating.data,Type == "all",Judgement == "fem"))
```

```
## boundary (singular) fit: see ?isSingular
```

```
summary(model_a1) %>% short(title = "Femininity Ratings - All Fertility")
```

```
## Warning in if (class(s) == "summary.clmm") {: the condition has length > 1 and
## only the first element will be used
```

Table 2: Femininity Ratings - All Fertility

	Estimate	Std. Error	df	t value	p value
(Intercept)	4.654	0.127	9.821	36.622	0.000
age	0.143	0.041	997.976	3.477	0.001
sex	-0.155	0.077	1027.236	-2.010	0.045
level	-0.051	0.057	4.489	-0.896	0.416

Textbook

```
model_a1 <- lmer(rating ~ age + sex + level +
                   (1 + age + sex + level || country_name) +
                   (1 + level || user_id),data = filter(rating.data,Type == "textbook",Judgement == "att
```

```
## boundary (singular) fit: see ?isSingular
```

```
summary(model_a1) %>% short(title = "Attractiveness Ratings - Textbook Fertility")
```

```
## Warning in if (class(s) == "summary.clmm") {: the condition has length > 1 and
## only the first element will be used
```

Table 3: Attractiveness Ratings - Textbook Fertility

	Estimate	Std. Error	df	t value	p value
(Intercept)	4.385	0.127	9.681	34.527	0.000
age	0.184	0.039	1043.070	4.759	0.000
sex	0.055	0.072	1064.719	0.766	0.444
level	0.040	0.050	4.090	0.791	0.472

```
model_a1 <- lmer(rating ~ age + sex + level +
                   (1 + age + sex + level || country_name) +
                   (1 + level || user_id),data = filter(rating.data,Type == "textbook",Judgement == "fem
```

```
## boundary (singular) fit: see ?isSingular
```

```
summary(model_a1) %>% short(title = "Femininity Ratings - Textbook Fertility")
```

```
## Warning in if (class(s) == "summary.clmm") {: the condition has length > 1 and
## only the first element will be used
```

Table 4: Femininity Ratings - Textbook Fertility

	Estimate	Std. Error	df	t value	p value
(Intercept)	4.696	0.116	10.530	40.532	0.000
age	0.132	0.042	976.588	3.153	0.002
sex	-0.153	0.123	1.943	-1.246	0.342
level	0.025	0.063	8.036	0.403	0.697

E

```
model_a1 <- lmer(rating ~ age + sex + level +
                  (1 + age + sex + level || country_name) +
                  (1 + level || user_id),data = filter(rating.data,Type == "E2",Judgement == "att"))
```

```
## boundary (singular) fit: see ?isSingular
```

```
## Warning: Model failed to converge with 1 negative eigenvalue: -1.1e-03
```

```
summary(model_a1) %>% short(title = "Attractiveness Ratings - Levels of E")
```

```
## Warning in if (class(s) == "summary.clmm") {: the condition has length > 1 and
## only the first element will be used
```

Table 5: Attractiveness Ratings - Levels of E

	Estimate	Std. Error	df	t value	p value
(Intercept)	4.301	0.115	9.697	37.523	0.000
age	0.146	0.060	2.410	2.429	0.114
sex	-0.083	0.073	1044.271	-1.139	0.255
level	0.112	0.032	1067.001	3.552	0.000

```
model_a1 <- lmer(rating ~ age + sex + level +
                  (1 + age + sex + level || country_name) +
                  (1 + level || user_id),data = filter(rating.data,Type == "E2",Judgement == "fem"))
```

```
## boundary (singular) fit: see ?isSingular
```

```
## Warning: Model failed to converge with 1 negative eigenvalue: -4.1e-03
```

```
summary(model_a1) %>% short(title = "Femininity Ratings - Levels of E")
```

```
## Warning in if (class(s) == "summary.clmm") {: the condition has length > 1 and
## only the first element will be used
```

Table 6: Femininity Ratings - Levels of E

	Estimate	Std. Error	df	t value	p value
(Intercept)	4.697	0.124	8.612	37.757	0.000
age	0.156	0.043	985.911	3.642	0.000
sex	-0.095	0.080	1025.005	-1.194	0.233
level	0.086	0.034	1030.003	2.545	0.011

P

```
model_a1 <- lmer(rating ~ age + sex + level +
                  (1 + age + sex + level || country_name) +
                  (1 + level || user_id),data = filter(rating.data,Type == "P",Judgement == "att"))
```

```
## boundary (singular) fit: see ?isSingular
```

```
summary(model_a1) %>% short(title = "Attractiveness Ratings - Levels of P")
```

```
## Warning in if (class(s) == "summary.clmm") {: the condition has length > 1 and
## only the first element will be used
```

Table 7: Attractiveness Ratings - Levels of P

	Estimate	Std. Error	df	t value	p value
(Intercept)	4.286	0.112	10.015	38.203	0.000
age	0.157	0.058	4.290	2.694	0.051
sex	-0.023	0.072	1050.016	-0.326	0.744
level	0.053	0.043	5.354	1.225	0.272

```
model_a1 <- lmer(rating ~ age + sex + level +
                  (1 + age + sex + level || country_name) +
                  (1 + level || user_id),data = filter(rating.data,Type == "P",Judgement == "fem"))
```

```
## boundary (singular) fit: see ?isSingular
```

```
## Warning: Model failed to converge with 1 negative eigenvalue: -1.4e-04
```

```
summary(model_a1) %>% short(title = "Femininity Ratings - Levels of P")
```

```
## Warning in if (class(s) == "summary.clmm") {: the condition has length > 1 and
## only the first element will be used
```

Table 8: Femininity Ratings - Levels of P

	Estimate	Std. Error	df	t value	p value
(Intercept)	4.635	0.111	9.374	41.651	0.000
age	0.159	0.043	969.230	3.654	0.000
sex	-0.152	0.082	1019.657	-1.865	0.063
level	-0.114	0.070	5.766	-1.630	0.156

EtoP

```
model_a1 <- lmer(rating ~ age + sex + level +
                  (1 + age + sex + level || country_name) +
                  (1 + level || user_id),data = filter(rating.data,Type == "EtoP",Judgement == "att"))
```

```
## boundary (singular) fit: see ?isSingular
```

```
summary(model_a1) %>% short(title = "Attractiveness Ratings - E to P Ratio")
```

```
## Warning in if (class(s) == "summary.clmm") {: the condition has length > 1 and
## only the first element will be used
```

Table 9: Attractiveness Ratings - E to P Ratio

	Estimate	Std. Error	df	t value	p value
(Intercept)	4.360	0.110	8.956	39.465	0.000
age	0.180	0.049	1.228	3.649	0.133
sex	-0.055	0.074	984.853	-0.743	0.458
level	0.053	0.033	1066.998	1.606	0.108

```
model_a1 <- lmer(rating ~ age + sex + level +
                  (1 + age + sex + level || country_name) +
                  (1 + level || user_id),data = filter(rating.data,Type == "EtoP",Judgement == "fem"))
```

```
## boundary (singular) fit: see ?isSingular
```

```
## Warning: Model failed to converge with 1 negative eigenvalue: -3.1e-04
```

```
summary(model_a1) %>% short(title = "Femininity Ratings - E to P Ratio")
```

```
## Warning in if (class(s) == "summary.clmm") {: the condition has length > 1 and
## only the first element will be used
```

Table 10: Femininity Ratings - E to P Ratio

	Estimate	Std. Error	df	t value	p value
(Intercept)	4.672	0.138	9.460	33.756	0.000
age	0.159	0.043	1002.724	3.729	0.000
sex	-0.152	0.080	1027.886	-1.905	0.057
level	-0.025	0.036	1030.000	-0.709	0.479

Are judgements of attractiveness and femininity for fertility cues consistent between tasks?

All

```
temp <- filter(afc.data, Type == "all",Judgement == "att") %>%
  select(user_id,choice,age,sex)
temp2 <- filter(rating.data,Type == "all",Judgement == "att") %>%
  select(user_id,country_name,level,rating) %>%
  mutate(level = recode(level,`-.5` = "low",`.5` = "high",`0` = "neutral")) %>%
  spread(key = level,value = rating) %>%
  mutate(difference = high - low)
temp3 <- left_join(temp2,temp,by = "user_id")

model <- lmer(difference ~ choice + age + sex +
                (1 + choice + age + sex || country_name),data = temp3)
```

```
## boundary (singular) fit: see ?isSingular
```

```
summary(model) %>% short(title = "Associations Between Tasks - All Fertility Attractiveness")
```

```
## Warning in if (class(s) == "summary.clmm") {: the condition has length > 1 and
## only the first element will be used
```

Table 11: Associations Between Tasks - All Fertility Attractiveness

	Estimate	Std. Error	df	t value	p value
(Intercept)	-0.052	0.035	1054	-1.484	0.138
choice	0.017	0.084	1054	0.202	0.840
age	-0.075	0.036	1054	-2.062	0.039
sex	0.091	0.070	1054	1.300	0.194

```
temp <- filter(afc.data, Type == "all",Judgement == "fem") %>%
  select(user_id,choice,age,sex)
temp2 <- filter(rating.data,Type == "all",Judgement == "fem") %>%
  select(user_id,country_name,level,rating) %>%
  mutate(level = recode(level,`-.5` = "low",`.5` = "high",`0` = "neutral")) %>%
  spread(key = level,value = rating) %>%
  mutate(difference = high - low)
temp3 <- left_join(temp2,temp,by = "user_id")

model <- lmer(difference ~ choice + age + sex +
                (1 + choice + age + sex || country_name),data = temp3)
```

```
## boundary (singular) fit: see ?isSingular
```

```
summary(model) %>% short(title = "Associations Between Tasks - All Fertility Femininity")
```

```
## Warning in if (class(s) == "summary.clmm") {: the condition has length > 1 and
## only the first element will be used
```

Table 12: Associations Between Tasks - All Fertility Femininity

	Estimate	Std. Error	df	t value	p value
(Intercept)	-0.060	0.054	4.073	-1.118	0.325
choice	0.230	0.086	2.621	2.687	0.086
age	0.028	0.058	8.689	0.492	0.635
sex	0.053	0.069	893.250	0.774	0.439

Textbook

```
temp <- filter(afc.data, Type == "textbook",Judgement == "att") %>%
  select(user_id,choice,age,sex)
temp2 <- filter(rating.data,Type == "textbook",Judgement == "att") %>%
  select(user_id,country_name,level,rating) %>%
  mutate(level = recode(level,`-.5` = "low",`.5` = "high",`0` = "neutral")) %>%
  spread(key = level,value = rating) %>%
  mutate(difference = high - low)
temp3 <- left_join(temp2,temp,by = "user_id")

model <- lmer(difference ~ choice + age + sex +
                (1 + choice + age + sex || country_name),data = temp3)
```

```
## boundary (singular) fit: see ?isSingular
```

```
summary(model) %>% short(title = "Associations Between Tasks - Textbook Fertility Attractiveness")
```

```
## Warning in if (class(s) == "summary.clmm") {: the condition has length > 1 and
## only the first element will be used
```

Table 13: Associations Between Tasks - Textbook Fertility Attractiveness

	Estimate	Std. Error	df	t value	p value
(Intercept)	0.028	0.051	4.062	0.538	0.618
choice	0.179	0.083	1049.783	2.166	0.031
age	0.014	0.044	4.147	0.320	0.764
sex	0.023	0.070	858.543	0.321	0.748

```
temp <- filter(afc.data, Type == "textbook",Judgement == "fem") %>%
  select(user_id,choice,age,sex)
temp2 <- filter(rating.data,Type == "textbook",Judgement == "fem") %>%
  select(user_id,country_name,level,rating) %>%
  mutate(level = recode(level,`-.5` = "low",`.5` = "high",`0` = "neutral")) %>%
  spread(key = level,value = rating) %>%
  mutate(difference = high - low)
temp3 <- left_join(temp2,temp,by = "user_id")

model <- lmer(difference ~ choice + age + sex +
                (1 + choice + age + sex || country_name),data = temp3)
```

```
## boundary (singular) fit: see ?isSingular
```

```
summary(model) %>% short(title = "Associations Between Tasks - Textbook Fertility Femininity")
```

```
## Warning in if (class(s) == "summary.clmm") {: the condition has length > 1 and
## only the first element will be used
```

Table 14: Associations Between Tasks - Textbook Fertility Femininity

	Estimate	Std. Error	df	t value	p value
(Intercept)	0.047	0.057	6.306	0.821	0.442
choice	0.039	0.088	1025.155	0.440	0.660
age	-0.081	0.041	1.302	-1.984	0.249
sex	0.045	0.073	824.258	0.615	0.538

E2

```
temp <- filter(afc.data, Type == "E2",Judgement == "att") %>%
  select(user_id,choice,age,sex)
temp2 <- filter(rating.data,Type == "E2",Judgement == "att") %>%
  select(user_id,country_name,level,rating) %>%
  mutate(level = recode(level,`-.5` = "low",`.5` = "high",`0` = "neutral")) %>%
  spread(key = level,value = rating) %>%
  mutate(difference = high - low)
temp3 <- left_join(temp2,temp,by = "user_id")
```

```
model <- lmer(difference ~ choice + age + sex +
                (1 + choice + age + sex || country_name),data = temp3)
```

```
## boundary (singular) fit: see ?isSingular
```

```
summary(model) %>% short(title = "Associations Between Tasks - Levels of E Attractiveness")
```

```
## Warning in if (class(s) == "summary.clmm") {: the condition has length > 1 and
## only the first element will be used
```

Table 15: Associations Between Tasks - Levels of E Attractiveness

	Estimate	Std. Error	df	t value	p value
(Intercept)	0.111	0.032	1054	3.460	0.001
choice	-0.050	0.077	1054	-0.649	0.516
age	-0.050	0.034	1054	-1.490	0.136
sex	-0.015	0.064	1054	-0.240	0.810

```
temp <- filter(afc.data, Type == "E2",Judgement == "fem") %>%
  select(user_id,choice,age,sex)
temp2 <- filter(rating.data,Type == "E2",Judgement == "fem") %>%
  select(user_id,country_name,level,rating) %>%
  mutate(level = recode(level,`-.5` = "low",`.5` = "high",`0` = "neutral")) %>%
  spread(key = level,value = rating) %>%
  mutate(difference = high - low)
temp3 <- left_join(temp2,temp,by = "user_id")

model <- lmer(difference ~ choice + age + sex +
                (1 + choice + age + sex || country_name),data = temp3)
```

```
## boundary (singular) fit: see ?isSingular
```

```
summary(model) %>% short(title = "Associations Between Tasks - Levels of E Femininity")
```

```
## Warning in if (class(s) == "summary.clmm") {: the condition has length > 1 and
## only the first element will be used
```

Table 16: Associations Between Tasks - Levels of E Femininity

	Estimate	Std. Error	df	t value	p value
(Intercept)	0.065	0.035	934.555	1.870	0.062
choice	0.165	0.123	2.569	1.341	0.286
age	0.041	0.036	1004.467	1.159	0.247
sex	0.036	0.118	5.203	0.305	0.772

P

```
temp <- filter(afc.data, Type == "P",Judgement == "att") %>%
  select(user_id,choice,age,sex)
temp2 <- filter(rating.data,Type == "P",Judgement == "att") %>%
  select(user_id,country_name,level,rating) %>%
  mutate(level = recode(level,`-.5` = "low",`.5` = "high",`0` = "neutral")) %>%
```

```
  spread(key = level,value = rating) %>%
  mutate(difference = high - low)
temp3 <- left_join(temp2,temp,by = "user_id")

model <- lmer(difference ~ choice + age + sex +
                (1 + choice + age + sex || country_name),data = temp3)
```

```
## boundary (singular) fit: see ?isSingular
```

```
summary(model) %>% short(title = "Associations Between Tasks - Levels of P Attractiveness")
```

```
## Warning in if (class(s) == "summary.clmm") {: the condition has length > 1 and
## only the first element will be used
```

Table 17: Associations Between Tasks - Levels of P Attractiveness

	Estimate	Std. Error	df	t value	p value
(Intercept)	0.051	0.042	4.147	1.208	0.292
choice	-0.085	0.082	1053.529	-1.032	0.302
age	-0.029	0.035	504.992	-0.811	0.418
sex	0.054	0.081	2.683	0.661	0.561

```
temp <- filter(afc.data, Type == "P",Judgement == "fem") %>%
  select(user_id,choice,age,sex)
temp2 <- filter(rating.data,Type == "P",Judgement == "fem") %>%
  select(user_id,country_name,level,rating) %>%
  mutate(level = recode(level,`-.5` = "low",`.5` = "high",`0` = "neutral")) %>%
  spread(key = level,value = rating) %>%
  mutate(difference = high - low)
temp3 <- left_join(temp2,temp,by = "user_id")

model <- lmer(difference ~ choice + age + sex +
                (1 + choice + age + sex || country_name),data = temp3)
```

```
## boundary (singular) fit: see ?isSingular
```

```
summary(model) %>% short(title = "Associations Between Tasks - Levels of P Femininity")
```

```
## Warning in if (class(s) == "summary.clmm") {: the condition has length > 1 and
## only the first element will be used
```

Table 18: Associations Between Tasks - Levels of P Femininity

	Estimate	Std. Error	df	t value	p value
(Intercept)	-0.105	0.058	4.342	-1.796	0.141
choice	0.093	0.087	1014.719	1.066	0.287
age	-0.003	0.038	673.695	-0.073	0.942
sex	0.237	0.072	895.378	3.276	0.001

EtoP

```
temp <- filter(afc.data, Type == "EtoP",Judgement == "att") %>%
  select(user_id,choice,age,sex)
```

```r
temp2 <- filter(rating.data,Type == "EtoP",Judgement == "att") %>%
  select(user_id,country_name,level,rating) %>%
  mutate(level = recode(level,`-.5` = "low",`.5` = "high",`0` = "neutral")) %>%
  spread(key = level,value = rating) %>%
  mutate(difference = high - low)
temp3 <- left_join(temp2,temp,by = "user_id")

model <- lmer(difference ~ choice + age + sex +
                (1 + choice + age + sex || country_name),data = temp3)
```

```
## boundary (singular) fit: see ?isSingular
```

```r
summary(model) %>% short(title = "Associations Between Tasks - E to P Ratio Attractiveness")
```

```
## Warning in if (class(s) == "summary.clmm") {: the condition has length > 1 and
## only the first element will be used
```

Table 19: Associations Between Tasks - E to P Ratio Attractiveness

	Estimate	Std. Error	df	t value	p value
(Intercept)	0.054	0.034	1053.985	1.608	0.108
choice	-0.057	0.082	2.177	-0.693	0.555
age	-0.029	0.035	1049.329	-0.835	0.404
sex	-0.034	0.068	1049.619	-0.503	0.615

```r
temp <- filter(afc.data, Type == "EtoP",Judgement == "fem") %>%
  select(user_id,choice,age,sex)
temp2 <- filter(rating.data,Type == "EtoP",Judgement == "fem") %>%
  select(user_id,country_name,level,rating) %>%
  mutate(level = recode(level,`-.5` = "low",`.5` = "high",`0` = "neutral")) %>%
  spread(key = level,value = rating) %>%
  mutate(difference = high - low)
temp3 <- left_join(temp2,temp,by = "user_id")

model <- lmer(difference ~ choice + age + sex +
                (1 + choice + age + sex || country_name),data = temp3)
```

```
## boundary (singular) fit: see ?isSingular
```

```r
summary(model) %>% short(title = "Associations Between Tasks - E to P Ratio Femininity")
```

```
## Warning in if (class(s) == "summary.clmm") {: the condition has length > 1 and
## only the first element will be used
```

Table 20: Associations Between Tasks - E to P Ratio Femininity

	Estimate	Std. Error	df	t value	p value
(Intercept)	-0.024	0.036	1026	-0.658	0.510
choice	0.172	0.086	1026	2.002	0.046
age	0.048	0.037	1026	1.278	0.202
sex	0.041	0.072	1026	0.578	0.564

Are associations between fertility cues and judgements of attractiveness and femininity moderated by individual differences?

3 Alternative Forced Choice Data

All

```
model1 <- clmm(as.factor(choice) ~ age + sex + sra + srh + financial_dificulties +
                  (1 + age + sex + sra + srh + financial_dificulties | country_name),data = filter(afc.d
```

Individual Differences Model

```
## Warning: Using formula(x) is deprecated when x is a character vector of length > 1.
##   Consider formula(paste(x, collapse = " ")) instead.
```

```
summary(model1) %>% short(title = "ID Model and Attractiveness 3afc - All Fertility")
```

Table 21: ID Model and Attractiveness 3afc - All Fertility

	Estimate	Std. Error	z value	p value
-0.5|0	-0.620	0.109	-5.706	0.000
0|0.5	0.688	0.107	6.400	0.000
age	-0.095	0.125	-0.762	0.446
sex	0.170	0.206	0.824	0.410
sra	-0.075	0.120	-0.627	0.531
srh	0.049	0.113	0.431	0.666
financial_dificulties	-0.046	0.087	-0.535	0.592

```
model16 <- clmm(as.factor(choice) ~ age + sex + sra + srh + financial_dificulties +
                  (1 + age + sex + sra + srh + financial_dificulties | country_name),data = filter(afc.d
```

```
## Warning: Using formula(x) is deprecated when x is a character vector of length > 1.
##   Consider formula(paste(x, collapse = " ")) instead.
```

```
summary(model16) %>% short(title = "ID Model and Femininity 3afc - All Fertility")
```

Table 22: ID Model and Femininity 3afc - All Fertility

	Estimate	Std. Error	z value	p value
-0.5|0	-0.669	0.113	-5.923	0.000
0|0.5	0.711	0.114	6.264	0.000
age	-0.129	0.067	-1.919	0.055
sex	0.167	0.121	1.378	0.168
sra	-0.049	0.071	-0.699	0.485
srh	0.050	0.075	0.666	0.505
financial_dificulties	-0.017	0.068	-0.244	0.807

```
model2 <- clmm(as.factor(choice) ~ age + sex + soi +
                  (1 + age + sex + soi | country_name),data = filter(afc.data, Type == "all",Judgement =
```

SOI

```
## Warning: Using formula(x) is deprecated when x is a character vector of length > 1.
##   Consider formula(paste(x, collapse = " ")) instead.
```

```
summary(model2) %>% short(title = "SOI Model and Attractiveness 3afc - All Fertility")
```

Table 23: SOI Model and Attractiveness 3afc - All Fertility

	Estimate	Std. Error	z value	p value
-0.5|0	-0.695	0.117	-5.935	0.000
0|0.5	0.787	0.121	6.504	0.000
age	-0.082	0.103	-0.797	0.425
sex	0.094	0.211	0.446	0.656
soi	-0.061	0.090	-0.674	0.500

```
model17 <- clmm(as.factor(choice) ~ age + sex + soi +
                  (1 + age + sex + soi | country_name),data = filter(afc.data, Type == "all",Judgement =
```

```
## Warning: Using formula(x) is deprecated when x is a character vector of length > 1.
##   Consider formula(paste(x, collapse = " ")) instead.
```

```
summary(model17) %>% short(title = "SOI Model and Femininity 3afc - All Fertility")
```

Table 24: SOI Model and Femininity 3afc - All Fertility

	Estimate	Std. Error	z value	p value
-0.5|0	-0.608	0.132	-4.598	0.000
0|0.5	0.734	0.135	5.453	0.000
age	-0.086	0.083	-1.034	0.301
sex	0.186	0.187	0.992	0.321
soi	-0.030	0.086	-0.347	0.728

Textbook

```
model4 <- clmm(as.factor(choice) ~ age + sex + sra + srh + financial_dificulties +
                  (1 + age + sex + sra + srh + financial_dificulties | country_name),data = filter(afc.
```

Individual Differences Model

```
## Warning: Using formula(x) is deprecated when x is a character vector of length > 1.
##   Consider formula(paste(x, collapse = " ")) instead.
```

```
summary(model4) %>% short(title = "ID Model and Attractiveness 3afc - Textbook Fertility")
```

Table 25: ID Model and Attractiveness 3afc - Textbook Fertility

	Estimate	Std. Error	z value	p value
-0.5|0	-0.815	0.110	-7.398	0.000
0|0.5	0.469	0.108	4.332	0.000
age	-0.136	0.147	-0.924	0.355
sex	0.307	0.266	1.153	0.249
sra	-0.168	0.133	-1.268	0.205
srh	0.063	0.107	0.593	0.553

	Estimate	Std. Error	z value	p value
financial_dificulties	-0.105	0.104	-1.007	0.314

```
model19 <- clmm(as.factor(choice) ~ age + sex + sra + srh + financial_dificulties +
                  (1 + age + sex + sra + srh + financial_dificulties | country_name),data = filter(afc.
```

```
## Warning: Using formula(x) is deprecated when x is a character vector of length > 1.
##   Consider formula(paste(x, collapse = " ")) instead.
```

```
summary(model19) %>% short(title = "ID Model and Femininity 3afc - Textbook Fertility")
```

Table 26: ID Model and Femininity 3afc - Textbook Fertility

	Estimate	Std. Error	z value	p value
-0.5|0	-0.749	0.083	-8.983	0.000
0|0.5	0.662	0.083	7.980	0.000
age	-0.101	0.140	-0.721	0.471
sex	0.015	0.142	0.103	0.918
sra	-0.081	0.088	-0.920	0.358
srh	0.079	0.106	0.749	0.454
financial_dificulties	0.107	0.079	1.344	0.179

```
model5 <- clmm(as.factor(choice) ~ age + sex + soi +
                 (1 + age + sex + soi | country_name),data = filter(afc.data, Type == "textbook",Judgem
```

SOI

```
## Warning: Using formula(x) is deprecated when x is a character vector of length > 1.
##   Consider formula(paste(x, collapse = " ")) instead.
```

```
summary(model5) %>% short(title = "SOI Model and Attractiveness 3afc - Textbook Fertility")
```

Table 27: SOI Model and Attractiveness 3afc - Textbook Fertility

	Estimate	Std. Error	z value	p value
-0.5|0	-0.866	0.097	-8.919	0.000
0|0.5	0.291	0.091	3.202	0.001
age	0.001	0.126	0.012	0.991
sex	0.400	0.233	1.714	0.086
soi	-0.032	0.101	-0.319	0.750

```
model20 <- clmm(as.factor(choice) ~ age + sex + soi +
                  (1 + soi | country_name),data = filter(afc.data, Type == "textbook",Judgement == "fem
summary(model20) %>% short(title = "SOI Model and Femininity 3afc - Textbook Fertility")
```

Table 28: SOI Model and Femininity 3afc - Textbook Fertility

	Estimate	Std. Error	z value	p value
-0.5|0	-0.642	0.149	-4.310	0.000
0|0.5	0.666	0.156	4.273	0.000

	Estimate	Std. Error	z value	p value
age	-0.001	0.082	-0.012	0.990
sex	-0.104	0.174	-0.598	0.550
soi	-0.058	0.091	-0.637	0.524

E2

```
model7 <- clmm(as.factor(choice) ~ age + sex + sra + srh + financial_dificulties +
                  (1 + age + sex + sra + srh + financial_dificulties | country_name),data = filter(afc.d
```

Individual Differences Model

```
## Warning: Using formula(x) is deprecated when x is a character vector of length > 1.
##   Consider formula(paste(x, collapse = " ")) instead.
```

```
summary(model7) %>% short(title = "ID Model and Attractiveness 3afc - Levels of E")
```

Table 29: ID Model and Attractiveness 3afc - Levels of E

	Estimate	Std. Error	z value	p value
-0.5|0	-0.600	0.070	-8.612	0.000
0|0.5	0.731	0.071	10.285	0.000
age	0.054	0.064	0.844	0.399
sex	-0.039	0.142	-0.276	0.783
sra	0.103	0.092	1.118	0.263
srh	-0.006	0.075	-0.077	0.938
financial_dificulties	0.023	0.095	0.245	0.806

```
model22 <- clmm(as.factor(choice) ~ age + sex + sra + srh + financial_dificulties +
                  (1 + age + sex + sra + srh + financial_dificulties | country_name),data = filter(afc.d
```

```
## Warning: Using formula(x) is deprecated when x is a character vector of length > 1.
##   Consider formula(paste(x, collapse = " ")) instead.
```

```
summary(model22) %>% short(title = "ID Model and Femininity 3afc - Levels of E")
```

Table 30: ID Model and Femininity 3afc - Levels of E

	Estimate	Std. Error	z value	p value
-0.5|0	-0.692	0.070	-9.883	0.000
0|0.5	0.692	0.070	9.877	0.000
age	-0.134	0.070	-1.929	0.054
sex	-0.300	0.141	-2.133	0.033
sra	-0.038	0.099	-0.386	0.700
srh	0.082	0.076	1.081	0.280
financial_dificulties	0.017	0.064	0.261	0.794

```
model8 <- clmm(as.factor(choice) ~ age + sex + soi +
                  (1 + age + sex + soi | country_name),data = filter(afc.data, Type == "E2",Judgement ==
```

SOI

```
## Warning: Using formula(x) is deprecated when x is a character vector of length > 1.
##   Consider formula(paste(x, collapse = " ")) instead.
```

```
summary(model8) %>% short(title = "SOI Model and Attractiveness 3afc - Levels of E")
```

Table 31: SOI Model and Attractiveness 3afc - Levels of E

	Estimate	Std. Error	z value	p value
-0.5|0	-0.660	0.100	-6.589	0.000
0|0.5	0.556	0.098	5.658	0.000
age	-0.009	0.084	-0.106	0.916
sex	-0.109	0.204	-0.536	0.592
soi	0.070	0.083	0.841	0.400

```
model23 <- clmm(as.factor(choice) ~ age + sex + soi +
                  (1 + soi | country_name),data = filter(afc.data, Type == "E2",Judgement == "fem"))
summary(model23) %>% short(title = "SOI Model and Femininity 3afc - Levels of E")
```

Table 32: SOI Model and Femininity 3afc - Levels of E

	Estimate	Std. Error	z value	p value
-0.5|0	-0.756	0.094	-8.054	0.000
0|0.5	0.564	0.091	6.188	0.000
age	-0.115	0.079	-1.455	0.146
sex	-0.267	0.161	-1.658	0.097
soi	0.024	0.080	0.307	0.759

P

```
model10 <- clmm(as.factor(choice) ~ age + sex + sra + srh + financial_dificulties +
                  (1 + age + sex + sra + srh + financial_dificulties | country_name),data = filter(afc.d
```

Individual Differences Model

```
## Warning: Using formula(x) is deprecated when x is a character vector of length > 1.
##   Consider formula(paste(x, collapse = " ")) instead.
```

```
summary(model10) %>% short(title = "ID Model and Attractiveness 3afc - Levels of P")
```

Table 33: ID Model and Attractiveness 3afc - Levels of P

	Estimate	Std. Error	z value	p value
-0.5|0	-0.644	0.087	-7.412	0.000
0|0.5	0.832	0.089	9.336	0.000
age	-0.118	0.099	-1.194	0.232
sex	-0.090	0.156	-0.578	0.563
sra	-0.168	0.103	-1.639	0.101
srh	0.133	0.105	1.264	0.206
financial_dificulties	-0.011	0.090	-0.119	0.905

```
model25 <- clmm(as.factor(choice) ~ age + sex + sra + srh + financial_dificulties +
                  (1 + age + sex + sra + srh + financial_dificulties | country_name),data = filter(afc.d
```

```
## Warning: Using formula(x) is deprecated when x is a character vector of length > 1.
##   Consider formula(paste(x, collapse = " ")) instead.
```

```
summary(model25) %>% short(title = "ID Model and Femininity 3afc - Levels of P")
```

Table 34: ID Model and Femininity 3afc - Levels of P

	Estimate	Std. Error	z value	p value
-0.5|0	-0.591	0.101	-5.867	0.000
0|0.5	0.811	0.104	7.823	0.000
age	-0.067	0.090	-0.747	0.455
sex	0.128	0.213	0.599	0.549
sra	-0.018	0.113	-0.161	0.872
srh	-0.008	0.102	-0.077	0.938
financial_dificulties	0.078	0.106	0.736	0.462

```
model11 <- clmm(as.factor(choice) ~ age + sex + soi +
                  (1 + age + sex + soi | country_name),data = filter(afc.data, Type == "P",Judgement ==
```

SOI

```
## Warning: Using formula(x) is deprecated when x is a character vector of length > 1.
##   Consider formula(paste(x, collapse = " ")) instead.
```

```
summary(model11) %>% short(title = "SOI Model and Attractiveness 3afc - Levels of P")
```

Table 35: SOI Model and Attractiveness 3afc - Levels of P

	Estimate	Std. Error	z value	p value
-0.5|0	-0.559	0.109	-5.118	0.000
0|0.5	0.786	0.112	7.006	0.000
age	-0.100	0.146	-0.684	0.494
sex	-0.050	0.180	-0.276	0.783
soi	-0.119	0.094	-1.265	0.206

```
model26 <- clmm(as.factor(choice) ~ age + sex + soi +
                  (1 + age+ sex + soi | country_name),data = filter(afc.data, Type == "P",Judgement ==
```

```
## Warning: Using formula(x) is deprecated when x is a character vector of length > 1.
##   Consider formula(paste(x, collapse = " ")) instead.
```

```
summary(model26) %>% short(title = "SOI Model and Femininity 3afc - Levels of P")
```

Table 36: SOI Model and Femininity 3afc - Levels of P

	Estimate	Std. Error	z value	p value
-0.5|0	-0.499	0.140	-3.565	0.000
0|0.5	0.879	0.146	6.026	0.000
age	-0.107	0.101	-1.054	0.292

	Estimate	Std. Error	z value	p value
sex	0.222	0.294	0.754	0.451
soi	-0.113	0.110	-1.029	0.304

EtoP

```
model13 <- clmm(as.factor(choice) ~ age + sex + sra + srh + financial_dificulties +
                  (1 + age + sex + sra + srh + financial_dificulties | country_name),data = filter(afc.
```

Individual Differences Model

```
## Warning: Using formula(x) is deprecated when x is a character vector of length > 1.
##   Consider formula(paste(x, collapse = " ")) instead.
```

```
summary(model13) %>% short(title = "ID Model and Attractiveness 3afc - E to P Ratio")
```

Table 37: ID Model and Attractiveness 3afc - E to P Ratio

	Estimate	Std. Error	z value	p value
-0.5|0	-0.594	0.076	-7.810	0.000
0|0.5	0.698	0.077	9.031	0.000
age	0.036	0.070	0.522	0.602
sex	-0.246	0.230	-1.068	0.286
sra	-0.071	0.073	-0.966	0.334
srh	0.008	0.082	0.100	0.921
financial_dificulties	0.083	0.083	1.001	0.317

```
model28 <- clmm(as.factor(choice) ~ age + sex + sra + srh + financial_dificulties +
                  (1 + age + sex + sra + srh + financial_dificulties | country_name),data = filter(afc.
```

```
## Warning: Using formula(x) is deprecated when x is a character vector of length > 1.
##   Consider formula(paste(x, collapse = " ")) instead.
```

```
summary(model28) %>% short(title = "ID Model and Femininity 3afc - E to P Ratio")
```

Table 38: ID Model and Femininity 3afc - E to P Ratio

	Estimate	Std. Error	z value	p value
-0.5|0	-0.496	0.097	-5.104	0.000
0|0.5	0.802	0.101	7.911	0.000
age	0.212	0.110	1.920	0.055
sex	-0.108	0.170	-0.636	0.525
sra	-0.119	0.111	-1.074	0.283
srh	0.010	0.102	0.099	0.921
financial_dificulties	0.035	0.088	0.393	0.694

```
model14 <- clmm(as.factor(choice) ~ age + sex + soi +
                  (1 + age + sex + soi | country_name),data = filter(afc.data, Type == "EtoP",Judgement
```

SOI

```
## Warning: Using formula(x) is deprecated when x is a character vector of length > 1.
##   Consider formula(paste(x, collapse = " ")) instead.
```

```
summary(model14) %>% short(title = "SOI Model and Attractiveness 3afc - E to P Ratio")
```

Table 39: SOI Model and Attractiveness 3afc - E to P Ratio

	Estimate	Std. Error	z value	p value
-0.5|0	-0.485	0.124	-3.917	0.000
0|0.5	0.607	0.125	4.863	0.000
age	0.011	0.117	0.091	0.927
sex	-0.179	0.322	-0.555	0.579
soi	0.047	0.094	0.504	0.614

```
model29 <- clmm(as.factor(choice) ~ age + sex + soi +
                  (1 + age + sex + soi | country_name),data = filter(afc.data, Type == "EtoP",Judgement
```

```
## Warning: Using formula(x) is deprecated when x is a character vector of length > 1.
##   Consider formula(paste(x, collapse = " ")) instead.
```

```
summary(model29) %>% short(title = "SOI Model and Femininity 3afc - E to P Ratio")
```

Table 40: SOI Model and Femininity 3afc - E to P Ratio

	Estimate	Std. Error	z value	p value
-0.5|0	-0.437	0.090	-4.867	0.000
0|0.5	0.695	0.093	7.473	0.000
age	0.157	0.077	2.042	0.041
sex	-0.040	0.162	-0.244	0.807
soi	-0.108	0.081	-1.330	0.183

Rating Data

All

```
model_a1 <- lmer(rating ~ age*level + sex*level + sra*level + srh*level + financial_dificulties*level +
                  (1 + age:level + sex:level + sra:level + srh:level + financial_dificulties:level || c
                  (1 + level || user_id),data = filter(rating.data,Type == "all",Judgement == "att"))
```

Individual Differences Model

```
## boundary (singular) fit: see ?isSingular
```

```
summary(model_a1) %>% short(title = "ID Model and Attractiveness Ratings - All Fertility")
```

```
## Warning in if (class(s) == "summary.clmm") {: the condition has length > 1 and
## only the first element will be used
```

Table 41: ID Model and Attractiveness Ratings - All Fertility

	Estimate	Std. Error	df	t value	p value
(Intercept)	4.306	0.112	9.850	38.316	0.000
age	0.188	0.038	1003.890	4.924	0.000

	Estimate	Std. Error	df	t value	p value
level	-0.067	0.036	907.458	-1.851	0.065
sex	-0.047	0.071	1034.352	-0.659	0.510
sra	0.021	0.042	1036.316	0.505	0.613
srh	0.115	0.042	1034.094	2.773	0.006
financial_dificulties	0.024	0.035	1036.604	0.667	0.505
age:level	-0.078	0.037	1029.032	-2.088	0.037
level:sex	0.089	0.071	1035.371	1.257	0.209
level:sra	-0.047	0.042	1035.869	-1.098	0.272
level:srh	0.034	0.060	9.904	0.560	0.588
level:financial_dificulties	-0.003	0.036	1032.671	-0.090	0.928

```
model_a16 <- lmer(rating ~ age*level + sex*level + sra*level + srh*level + financial_dificulties*level +
                    (1 + age:level + sex:level + sra:level + srh:level + financial_dificulties:level ||
                    (1 + level || user_id),data = filter(rating.data,Type == "all",Judgement == "fem"))
```

```
## boundary (singular) fit: see ?isSingular
```

```
summary(model_a16) %>% short(title = "ID Model and Femininity Ratings - All Fertility")
```

```
## Warning in if (class(s) == "summary.clmm") {: the condition has length > 1 and
## only the first element will be used
```

Table 42: ID Model and Femininity Ratings - All Fertility

	Estimate	Std. Error	df	t value	p value
(Intercept)	4.660	0.127	9.892	36.694	0.000
age	0.143	0.042	969.848	3.421	0.001
level	-0.008	0.037	885.659	-0.211	0.833
sex	-0.140	0.078	1000.194	-1.802	0.072
sra	-0.005	0.046	1000.747	-0.098	0.922
srh	0.130	0.045	997.499	2.866	0.004
financial_dificulties	0.014	0.039	1000.965	0.369	0.712
age:level	0.009	0.058	8.554	0.161	0.876
level:sex	0.069	0.071	1910.073	0.977	0.329
level:sra	0.005	0.066	5.344	0.081	0.938
level:srh	0.063	0.042	2000.452	1.503	0.133
level:financial_dificulties	0.032	0.036	1532.439	0.907	0.365

```
model_a2 <- lmer(rating ~ age*level + sex*level + soi*level +
                   (1 + age:level + sex:level + soi:level || country_name) +
                   (1 + level || user_id),data = filter(rating.data,Type == "all",Judgement == "att"))
```

SOI Model

```
## boundary (singular) fit: see ?isSingular
```

```
summary(model_a2) %>% short(title = "SOI Model and Attractiveness Ratings - All Fertility")
```

```
## Warning in if (class(s) == "summary.clmm") {: the condition has length > 1 and
## only the first element will be used
```

Table 43: SOI Model and Attractiveness Ratings - All Fertility

	Estimate	Std. Error	df	t value	p value
(Intercept)	4.246	0.135	8.781	31.466	0.000
age	0.149	0.044	523.325	3.402	0.001
level	0.020	0.045	270.583	0.448	0.654
sex	-0.207	0.092	534.358	-2.240	0.026
soi	0.061	0.044	534.993	1.380	0.168
age:level	-0.092	0.043	534.663	-2.141	0.033
level:sex	0.118	0.105	2.787	1.128	0.347
level:soi	-0.013	0.044	525.577	-0.302	0.763

```
model_a17 <- lmer(rating ~ age*level + sex*level + soi*level +
                  (1 + age:level + sex:level + soi:level || country_name) +
                  (1 + level || user_id),data = filter(rating.data,Type == "all",Judgement == "fem"))
```

```
## boundary (singular) fit: see ?isSingular
```

```
summary(model_a17) %>% short(title = "SOI Model and Femininity Ratings - All Fertility")
```

```
## Warning in if (class(s) == "summary.clmm") {: the condition has length > 1 and
## only the first element will be used
```

Table 44: SOI Model and Femininity Ratings - All Fertility

	Estimate	Std. Error	df	t value	p value
(Intercept)	4.552	0.122	6.679	37.453	0.000
age	0.186	0.047	508.516	3.998	0.000
level	-0.114	0.043	442.302	-2.658	0.008
sex	-0.388	0.098	527.020	-3.950	0.000
soi	0.069	0.047	533.789	1.483	0.139
age:level	0.040	0.053	7.290	0.750	0.477
level:sex	0.061	0.085	522.122	0.718	0.473
level:soi	-0.005	0.054	6.599	-0.097	0.926

Textbook

```
model_a4 <- lmer(rating ~ age*level + sex*level + sra*level + srh*level + financial_dificulties*level +
                  (1 + age:level + sex:level + sra:level + srh:level + financial_dificulties:level || c
                  (1 + level || user_id),data = filter(rating.data,Type == "textbook",Judgement == "at
```

Individual Differences Model

```
## boundary (singular) fit: see ?isSingular
```

```
summary(model_a4) %>% short(title = "ID Model and Attractiveness Ratings - Textbook Fertility")
```

```
## Warning in if (class(s) == "summary.clmm") {: the condition has length > 1 and
## only the first element will be used
```

Table 45: ID Model and Attractiveness Ratings - Textbook Fertility

	Estimate	Std. Error	df	t value	p value
(Intercept)	4.388	0.127	9.885	34.663	0.000
age	0.183	0.039	1014.138	4.671	0.000
level	0.087	0.036	872.229	2.413	0.016
sex	0.077	0.073	1036.448	1.052	0.293
sra	0.035	0.043	1036.991	0.802	0.423
srh	0.060	0.043	1032.937	1.420	0.156
financial_dificulties	-0.001	0.036	1036.986	-0.040	0.968
age:level	0.004	0.037	1036.965	0.100	0.920
level:sex	0.031	0.071	1036.414	0.442	0.659
level:sra	0.007	0.042	1030.434	0.165	0.869
level:srh	0.007	0.042	1036.824	0.173	0.863
level:financial_dificulties	0.008	0.049	5.784	0.158	0.880

```
model_a19 <- lmer(rating ~ age*level + sex*level + sra*level + srh*level + financial_dificulties*level +
                    (1 + age:level + sex:level + sra:level + srh:level + financial_dificulties:level ||
                    (1 + level || user_id),data = filter(rating.data,Type == "textbook",Judgement == "fe
```

```
## boundary (singular) fit: see ?isSingular
```

```
## Warning: Model failed to converge with 1 negative eigenvalue: -2.0e+02
```

```
summary(model_a19) %>% short(title = "ID Model and Femininity Ratings - Textbook Fertility")
```

```
## Warning in if (class(s) == "summary.clmm") {: the condition has length > 1 and
## only the first element will be used
```

Table 46: ID Model and Femininity Ratings - Textbook Fertility

	Estimate	Std. Error	df	t value	p value
(Intercept)	4.703	0.117	10.474	40.337	0.000
age	0.125	0.042	960.326	2.968	0.003
level	0.094	0.037	622.672	2.509	0.012
sex	-0.058	0.078	997.819	-0.734	0.463
sra	-0.012	0.047	999.178	-0.254	0.800
srh	0.129	0.046	998.769	2.814	0.005
financial_dificulties	-0.004	0.039	1000.429	-0.113	0.910
age:level	-0.109	0.039	999.501	-2.809	0.005
level:sex	0.059	0.074	996.824	0.801	0.423
level:sra	-0.020	0.044	990.494	-0.459	0.646
level:srh	-0.049	0.044	1000.881	-1.112	0.266
level:financial_dificulties	-0.053	0.041	2.700	-1.297	0.294

```
model_a5 <- lmer(rating ~ age*level + sex*level + soi*level +
                   (1 + age:level + sex:level + soi:level || country_name) +
                   (1 + level || user_id),data = filter(rating.data,Type == "textbook",Judgement == "att
```

SOI Model

```
## boundary (singular) fit: see ?isSingular
```

```
summary(model_a5) %>% short(title = "SOI Model and Attractiveness Ratings - Textbook Fertility")
```

```
## Warning in if (class(s) == "summary.clmm") {: the condition has length > 1 and
## only the first element will be used
```

Table 47: SOI Model and Attractiveness Ratings - Textbook Fertility

	Estimate	Std. Error	df	t value	p value
(Intercept)	4.275	0.151	8.533	28.229	0.000
age	0.172	0.046	526.774	3.768	0.000
level	-0.002	0.040	530.294	-0.044	0.965
sex	-0.141	0.096	534.971	-1.475	0.141
soi	0.077	0.046	534.870	1.683	0.093
age:level	-0.001	0.038	534.950	-0.035	0.972
level:sex	-0.068	0.079	522.201	-0.863	0.389
level:soi	0.021	0.042	5.613	0.511	0.629

```
model_a20 <- lmer(rating ~ age*level + sex*level + soi*level +
                  (1 + age:level + sex:level + soi:level || country_name) +
                  (1 + level || user_id),data = filter(rating.data,Type == "textbook",Judgement == "fem
```

```
## boundary (singular) fit: see ?isSingular
```

```
summary(model_a20) %>% short(title = "SOI Model and Femininity Ratings - Textbook Fertility")
```

```
## Warning in if (class(s) == "summary.clmm") {: the condition has length > 1 and
## only the first element will be used
```

Table 48: SOI Model and Femininity Ratings - Textbook Fertility

	Estimate	Std. Error	df	t value	p value
(Intercept)	4.629	0.098	5.342	47.418	0.000
age	0.134	0.048	487.792	2.815	0.005
level	-0.028	0.042	430.808	-0.661	0.509
sex	-0.325	0.101	485.225	-3.227	0.001
soi	0.090	0.048	527.325	1.877	0.061
age:level	-0.047	0.052	6.627	-0.912	0.394
level:sex	-0.037	0.083	513.087	-0.442	0.658
level:soi	-0.028	0.043	7.027	-0.659	0.531

E Model

```
model_a7 <- lmer(rating ~ age*level + sex*level + sra*level + srh*level + financial_dificulties*level +
                 (1 + age:level + sex:level + sra:level + srh:level + financial_dificulties:level || 
                 (1 + level || user_id),data = filter(rating.data,Type == "E2",Judgement == "att"))
```

Individual Differences

```
## boundary (singular) fit: see ?isSingular
```

```
## Warning: Model failed to converge with 1 negative eigenvalue: -3.3e-04
```

```
summary(model_a7) %>% short(title = "ID Model and Attractiveness Ratings - Levels of E")
```

```
## Warning in if (class(s) == "summary.clmm") {: the condition has length > 1 and
## only the first element will be used
```

Table 49: ID Model and Attractiveness Ratings - Levels of E

	Estimate	Std. Error	df	t value	p value
(Intercept)	4.286	0.115	9.755	37.145	0.000
age	0.188	0.039	1003.749	4.795	0.000
level	0.108	0.033	721.262	3.226	0.001
sex	-0.073	0.073	1034.379	-0.998	0.318
sra	-0.011	0.043	1036.336	-0.262	0.793
srh	0.131	0.043	1034.056	3.065	0.002
financial_dificulties	0.029	0.036	1036.616	0.799	0.424
age:level	-0.052	0.034	1017.007	-1.507	0.132
level:sex	-0.010	0.066	1031.851	-0.157	0.876
level:sra	0.001	0.039	1033.464	0.030	0.976
level:srh	-0.014	0.054	3.801	-0.263	0.806
level:financial_dificulties	-0.020	0.033	1025.254	-0.597	0.551

```
model_a22 <- lmer(rating ~ age*level + sex*level + sra*level + srh*level + financial_dificulties*level +
                    (1 + age:level + sex:level + sra:level + srh:level + financial_dificulties:level ||
                    (1 + level || user_id),data = filter(rating.data,Type == "E2",Judgement == "fem"))
```

```
## boundary (singular) fit: see ?isSingular
```

```
## Warning: Model failed to converge with 1 negative eigenvalue: -2.5e-03
```

```
summary(model_a22) %>% short(title = "ID Model and Femininity Ratings - Levels of E")
```

```
## Warning in if (class(s) == "summary.clmm") {: the condition has length > 1 and
## only the first element will be used
```

Table 50: ID Model and Femininity Ratings - Levels of E

	Estimate	Std. Error	df	t value	p value
(Intercept)	4.720	0.127	8.713	37.262	0.000
age	0.153	0.043	960.430	3.542	0.000
level	0.062	0.035	933.603	1.758	0.079
sex	-0.083	0.081	999.164	-1.034	0.302
sra	-0.029	0.048	1000.171	-0.600	0.549
srh	0.121	0.047	997.568	2.558	0.011
financial_dificulties	0.010	0.040	1000.792	0.260	0.795
age:level	0.035	0.037	990.617	0.953	0.341
level:sex	0.025	0.129	6.332	0.191	0.855
level:sra	0.029	0.042	1000.106	0.696	0.486
level:srh	0.000	0.042	998.094	-0.011	0.991
level:financial_dificulties	0.010	0.035	1000.704	0.273	0.785

```
model_a8 <- lmer(rating ~ age*level + sex*level + soi*level +
```

```
                   (1 + age:level + sex:level + soi:level || country_name) +
                   (1 + level || user_id),data = filter(rating.data,Type == "E2",Judgement == "att"))
```

SOI Model

```
## boundary (singular) fit: see ?isSingular
```

```
summary(model_a8) %>% short(title = "SOI Model and Attractiveness Ratings - Levels of E")
```

```
## Warning in if (class(s) == "summary.clmm") {: the condition has length > 1 and
## only the first element will be used
```

Table 51: SOI Model and Attractiveness Ratings - Levels of E

	Estimate	Std. Error	df	t value	p value
(Intercept)	4.242	0.136	9.088	31.202	0.000
age	0.134	0.044	523.996	3.055	0.002
level	0.086	0.039	533.458	2.181	0.030
sex	-0.209	0.092	534.459	-2.258	0.024
soi	0.052	0.044	534.999	1.171	0.242
age:level	-0.019	0.038	534.740	-0.508	0.611
level:sex	-0.057	0.079	533.111	-0.723	0.470
level:soi	-0.092	0.052	8.790	-1.759	0.113

```
model_a23 <- lmer(rating ~ age*level + sex*level + soi*level +
                   (1 + age:level + sex:level + soi:level || country_name) +
                   (1 + level || user_id),data = filter(rating.data,Type == "E2",Judgement == "fem"))
```

```
## boundary (singular) fit: see ?isSingular
```

```
summary(model_a23) %>% short(title = "SOI Model and Femininity Ratings - Levels of E")
```

```
## Warning in if (class(s) == "summary.clmm") {: the condition has length > 1 and
## only the first element will be used
```

Table 52: SOI Model and Femininity Ratings - Levels of E

	Estimate	Std. Error	df	t value	p value
(Intercept)	4.608	0.123	6.327	37.456	0.000
age	0.191	0.047	506.741	4.039	0.000
level	0.069	0.042	342.944	1.643	0.101
sex	-0.304	0.100	526.168	-3.046	0.002
soi	0.037	0.048	533.651	0.769	0.442
age:level	-0.008	0.040	7.111	-0.199	0.848
level:sex	-0.032	0.136	7.085	-0.237	0.819
level:soi	0.048	0.040	534.838	1.205	0.229

P Model

```
model_a10 <- lmer(rating ~ age*level + sex*level + sra*level + srh*level + financial_dificulties*level +
                     (1 + age:level + sex:level + sra:level + srh:level + financial_dificulties:level ||
                     (1 + level || user_id),data = filter(rating.data,Type == "P",Judgement == "att"))
```

Individual Differences Model

```
## boundary (singular) fit: see ?isSingular
```

```
summary(model_a10) %>% short(title = "ID Model and Attractiveness Ratings - Levels of P")
```

```
## Warning in if (class(s) == "summary.clmm") {: the condition has length > 1 and
## only the first element will be used
```

Table 53: ID Model and Attractiveness Ratings - Levels of P

	Estimate	Std. Error	df	t value	p value
(Intercept)	4.274	0.115	9.780	37.327	0.000
age	0.202	0.039	1003.737	5.186	0.000
level	0.069	0.035	716.770	1.979	0.048
sex	-0.017	0.073	1034.359	-0.239	0.811
sra	-0.016	0.043	1036.326	-0.369	0.712
srh	0.088	0.042	1034.072	2.087	0.037
financial_dificulties	0.035	0.036	1036.612	0.983	0.326
age:level	-0.045	0.036	946.523	-1.267	0.205
level:sex	0.049	0.084	2.668	0.583	0.606
level:sra	-0.041	0.041	1018.884	-1.008	0.314
level:srh	0.003	0.040	1007.282	0.072	0.943
level:financial_dificulties	0.022	0.049	5.919	0.458	0.663

```
model_a25 <- lmer(rating ~ age*level + sex*level + sra*level + srh*level + financial_dificulties*level +
                    (1 + age:level + sex:level + sra:level + srh:level + financial_dificulties:level ||
                    (1 + level || user_id),data = filter(rating.data,Type == "P",Judgement == "fem"))
```

```
## boundary (singular) fit: see ?isSingular
```

```
## Warning: Model failed to converge with 1 negative eigenvalue: -3.5e-03
```

```
summary(model_a25) %>% short(title = "ID Model and Femininity Ratings - Levels of P")
```

```
## Warning in if (class(s) == "summary.clmm") {: the condition has length > 1 and
## only the first element will be used
```

Table 54: ID Model and Femininity Ratings - Levels of P

	Estimate	Std. Error	df	t value	p value
(Intercept)	4.645	0.112	9.172	41.555	0.000
age	0.157	0.044	942.276	3.576	0.000
level	-0.062	0.037	498.986	-1.676	0.094
sex	-0.131	0.082	992.754	-1.601	0.110
sra	0.000	0.049	995.347	0.008	0.994
srh	0.156	0.048	999.487	3.248	0.001
financial_dificulties	0.024	0.041	999.076	0.597	0.550
age:level	-0.012	0.038	658.572	-0.327	0.744
level:sex	0.274	0.086	0.571	3.203	0.322
level:sra	-0.034	0.043	947.394	-0.778	0.437
level:srh	0.017	0.043	890.057	0.382	0.703
level:financial_dificulties	-0.003	0.045	4.089	-0.078	0.942

```
model_a11 <- lmer(rating ~ age*level + sex*level + soi*level +
                  (1 + age:level + sex:level + soi:level || country_name) +
                  (1 + level || user_id),data = filter(rating.data,Type == "P",Judgement == "att"))
```

SOI Model

```
## Warning in checkConv(attr(opt, "derivs"), opt$par, ctrl = control$checkConv, :
## unable to evaluate scaled gradient

## Warning in checkConv(attr(opt, "derivs"), opt$par, ctrl = control$checkConv, :
## Model failed to converge: degenerate Hessian with 1 negative eigenvalues
```

```
summary(model_a11) %>% short(title = "SOI Model and Attractiveness Ratings - Levels of P")
```

```
## Warning in if (class(s) == "summary.clmm") {: the condition has length > 1 and
## only the first element will be used
```

Table 55: SOI Model and Attractiveness Ratings - Levels of P

	Estimate	Std. Error	df	t value	p value
(Intercept)	4.233	0.129	9.656	32.926	0.000
age	0.143	0.044	521.880	3.256	0.001
level	-0.040	0.040	305.421	-0.996	0.320
sex	-0.161	0.093	533.492	-1.733	0.084
soi	0.039	0.044	534.893	0.879	0.380
age:level	-0.045	0.044	4.334	-1.023	0.360
level:sex	0.109	0.103	5.614	1.054	0.335
level:soi	-0.054	0.073	7.884	-0.746	0.477

```
model_a26 <- lmer(rating ~ age*level + sex*level + soi*level +
                  (1 + age:level + sex:level + soi:level || country_name) +
                  (1 + level || user_id),data = filter(rating.data,Type == "P",Judgement == "fem"))
```

```
## boundary (singular) fit: see ?isSingular
```

```
summary(model_a26) %>% short(title = "SOI Model and Femininity Ratings - Levels of P")
```

```
## Warning in if (class(s) == "summary.clmm") {: the condition has length > 1 and
## only the first element will be used
```

Table 56: SOI Model and Femininity Ratings - Levels of P

	Estimate	Std. Error	df	t value	p value
(Intercept)	4.587	0.089	5.978	51.521	0.000
age	0.153	0.047	490.592	3.216	0.001
level	-0.109	0.048	304.656	-2.290	0.023
sex	-0.339	0.100	469.723	-3.385	0.001
soi	0.010	0.048	525.816	0.206	0.837
age:level	-0.003	0.045	534.678	-0.073	0.942
level:sex	0.182	0.121	2.971	1.504	0.231
level:soi	0.043	0.066	3.071	0.655	0.558

E/P Model

```
model_a13 <- lmer(rating ~ age*level + sex*level + sra*level + srh*level + financial_dificulties*level +
                    (1 + age:level + sex:level + sra:level + srh:level + financial_dificulties:level ||
                    (1 + level || user_id),data = filter(rating.data,Type == "EtoP",Judgement == "att"))
```

Individual Differences

```
## boundary (singular) fit: see ?isSingular
```

```
summary(model_a13) %>% short(title = "ID Model and Attractiveness Ratings - E to P Ratio")
```

```
## Warning in if (class(s) == "summary.clmm") {: the condition has length > 1 and
## only the first element will be used
```

Table 57: ID Model and Attractiveness Ratings - E to P Ratio

	Estimate	Std. Error	df	t value	p value
(Intercept)	4.353	0.110	8.964	39.473	0.000
age	0.197	0.040	992.779	4.967	0.000
level	0.061	0.035	568.675	1.762	0.079
sex	-0.026	0.074	1031.469	-0.350	0.726
sra	0.028	0.044	1034.924	0.633	0.527
srh	0.126	0.043	1034.592	2.906	0.004
financial_dificulties	0.059	0.037	1035.941	1.600	0.110
age:level	-0.028	0.036	1015.069	-0.773	0.439
level:sex	-0.044	0.068	1031.017	-0.637	0.524
level:sra	0.091	0.057	2.871	1.595	0.213
level:srh	-0.055	0.041	1036.693	-1.345	0.179
level:financial_dificulties	-0.003	0.034	988.649	-0.086	0.932

```
model_a28 <- lmer(rating ~ age*level + sex*level + sra*level + srh*level + financial_dificulties*level +
                    (1 + age:level + sex:level + sra:level + srh:level + financial_dificulties:level ||
                    (1 + level || user_id),data = filter(rating.data,Type == "EtoP",Judgement == "fem"))
```

```
## boundary (singular) fit: see ?isSingular
```

```
## Warning: Model failed to converge with 1 negative eigenvalue: -1.2e-03
```

```
summary(model_a28) %>% short(title = "ID Model and Femininity Ratings - E to P Ratio")
```

```
## Warning in if (class(s) == "summary.clmm") {: the condition has length > 1 and
## only the first element will be used
```

Table 58: ID Model and Femininity Ratings - E to P Ratio

	Estimate	Std. Error	df	t value	p value
(Intercept)	4.683	0.139	9.609	33.773	0.000
age	0.159	0.043	974.551	3.673	0.000
level	-0.028	0.037	1001.000	-0.760	0.447
sex	-0.142	0.081	1000.773	-1.765	0.078
sra	0.001	0.048	1000.987	0.017	0.986
srh	0.108	0.047	996.765	2.289	0.022
financial_dificulties	0.034	0.040	1000.992	0.836	0.403
age:level	0.048	0.038	1001.000	1.248	0.212
level:sex	0.031	0.073	1000.967	0.429	0.668

	Estimate	Std. Error	df	t value	p value
level:sra	0.032	0.044	1001.000	0.734	0.463
level:srh	-0.024	0.044	1001.000	-0.542	0.588
level:financial_dificulties	-0.005	0.037	1001.000	-0.143	0.886

```
model_a14 <- lmer(rating ~ age*level + sex*level + soi*level +
                  (1 + age:level + sex:level + soi:level || country_name) +
                  (1 + level || user_id),data = filter(rating.data,Type == "EtoP",Judgement == "att"))
```

SOI Model

```
## boundary (singular) fit: see ?isSingular
```

```
## Warning: Model failed to converge with 1 negative eigenvalue: -2.1e-03
```

```
summary(model_a14) %>% short(title = "SOI Model and Attractiveness Ratings - E to P Ratio")
```

```
## Warning in if (class(s) == "summary.clmm") {: the condition has length > 1 and
## only the first element will be used
```

Table 59: SOI Model and Attractiveness Ratings - E to P Ratio

	Estimate	Std. Error	df	t value	p value
(Intercept)	4.291	0.128	8.327	33.627	0.000
age	0.151	0.044	518.852	3.402	0.001
level	0.089	0.039	535.001	2.309	0.021
sex	-0.203	0.094	532.759	-2.168	0.031
soi	0.070	0.045	534.794	1.575	0.116
age:level	-0.108	0.037	535.001	-2.911	0.004
level:sex	-0.104	0.077	535.001	-1.348	0.178
level:soi	0.038	0.038	535.001	0.993	0.321

```
model_a29 <- lmer(rating ~ age*level + sex*level + soi*level +
                  (1 + age:level + sex:level + soi:level || country_name) +
                  (1 + level || user_id),data = filter(rating.data,Type == "EtoP",Judgement == "fem"))
```

```
## boundary (singular) fit: see ?isSingular
```

```
summary(model_a29) %>% short(title = "SOI Model and Femininity Ratings - E to P Ratio")
```

```
## Warning in if (class(s) == "summary.clmm") {: the condition has length > 1 and
## only the first element will be used
```

Table 60: SOI Model and Femininity Ratings - E to P Ratio

	Estimate	Std. Error	df	t value	p value
(Intercept)	4.588	0.138	7.661	33.318	0.000
age	0.184	0.047	518.230	3.885	0.000
level	0.003	0.042	534.999	0.077	0.938
sex	-0.360	0.100	532.960	-3.603	0.000
soi	0.031	0.048	534.845	0.658	0.511
age:level	0.001	0.040	534.999	0.033	0.974
level:sex	0.043	0.083	534.998	0.516	0.606

	Estimate	Std. Error	df	t value	p value
level:soi	0.074	0.041	534.999	1.816	0.070

Do country factors moderate the association between attractiveness and femininity judgements and cues to fertility?

All

3afc

```
model3 <- clmm(as.factor(choice) ~ health.fa + inequality.fa +
                  (1 | country_name) +
                  (1 + health.fa + inequality.fa | region),data = filter(afc.data,Type == "all",Judgemen
```

```
## Warning: Using formula(x) is deprecated when x is a character vector of length > 1.
##   Consider formula(paste(x, collapse = " ")) instead.
```

```
summary(model3) %>% short(title = "Ecological Factors and Attractiveness 3afc - All Fertility")
```

Table 61: Ecological Factors and Attractiveness 3afc - All Fertility

	Estimate	Std. Error	z value	p value
-0.5|0	-0.637	0.099	-6.432	0.000
0|0.5	0.644	0.099	6.500	0.000
health.fa	0.029	0.101	0.288	0.773
inequality.fa	0.006	0.062	0.100	0.921

```
model18 <- clmm(as.factor(choice) ~ health.fa + inequality.fa +
                  (1 | country_name) +
                  (1 + health.fa + inequality.fa | region),data = filter(afc.data,Type == "all",Judgemen
```

```
## Warning: Using formula(x) is deprecated when x is a character vector of length > 1.
##   Consider formula(paste(x, collapse = " ")) instead.
```

```
summary(model18) %>% short(title = "Ecological Factors and Femininity 3afc - All Fertility")
```

Table 62: Ecological Factors and Femininity 3afc - All Fertility

	Estimate	Std. Error	z value	p value
-0.5|0	-0.656	0.164	-3.997	0.000
0|0.5	0.749	0.165	4.545	0.000
health.fa	-0.159	0.119	-1.330	0.184
inequality.fa	0.074	0.078	0.941	0.347

Rating

```
model_a3 <- lmer(rating ~ health.fa*level + inequality.fa*level +
                  (1 + level || country_name) +
                  (1 + health.fa:level + inequality.fa:level || region) +
                  (1 + health.fa:level + inequality.fa:level || user_id),data = filter(rating.data,Type
```

```
## boundary (singular) fit: see ?isSingular
```

```
summary(model_a3) %>% short(title = "Ecological Factors and Attractiveness Ratings - All Fertility")
```

```
## Warning in if (class(s) == "summary.clmm") {: the condition has length > 1 and
## only the first element will be used
```

Table 63: Ecological Factors and Attractiveness Ratings - All Fertility

	Estimate	Std. Error	df	t value	p value
(Intercept)	4.373	0.099	9.061	44.149	0.000
health.fa	-0.040	0.099	8.342	-0.404	0.697
level	-0.071	0.058	95.248	-1.226	0.223
inequality.fa	-0.010	0.092	5.308	-0.113	0.914
health.fa:level	-0.093	0.103	1.373	-0.900	0.498
level:inequality.fa	-0.004	0.068	1.412	-0.060	0.960

```
model_a18 <- lmer(rating ~ health.fa*level + inequality.fa*level +
                  (1 + level || country_name) +
                  (1 + health.fa:level + inequality.fa:level || region) +
                  (1 + health.fa:level + inequality.fa:level || user_id),data = filter(rating.data,Type
```

```
## boundary (singular) fit: see ?isSingular
```

```
summary(model_a18) %>% short(title = "Ecological Factors and Femininity Ratings - All Fertility")
```

```
## Warning in if (class(s) == "summary.clmm") {: the condition has length > 1 and
## only the first element will be used
```

Table 64: Ecological Factors and Femininity Ratings - All Fertility

	Estimate	Std. Error	df	t value	p value
(Intercept)	4.657	0.137	8.348	34.040	0.000
health.fa	-0.019	0.138	7.164	-0.140	0.893
level	-0.082	0.056	1634.897	-1.468	0.142
inequality.fa	-0.020	0.134	5.962	-0.146	0.888
health.fa:level	-0.062	0.062	1525.079	-1.002	0.317
level:inequality.fa	-0.043	0.039	1927.701	-1.118	0.264

Textbook

3afc

```
model6 <- clmm(as.factor(choice) ~ health.fa + inequality.fa +
                  (1 | country_name) +
                  (1 + health.fa + inequality.fa | region),data = filter(afc.data,Type == "textbook",Ju
```

```
## Warning: Using formula(x) is deprecated when x is a character vector of length > 1.
##   Consider formula(paste(x, collapse = " ")) instead.
```

```
summary(model6) %>% short(title = "Ecological Factors and Attractiveness 3afc - Textbook Fertility")
```

Table 65: Ecological Factors and Attractiveness 3afc - Textbook Fertility

	Estimate	Std. Error	z value	p value
-0.5|0	-0.791	0.171	-4.619	0.000
0|0.5	0.472	0.170	2.777	0.005
health.fa	-0.328	0.184	-1.779	0.075
inequality.fa	0.169	0.124	1.358	0.175

```
model21 <- clmm(as.factor(choice) ~ health.fa + inequality.fa +
                  (1 | country_name) +
                  (1 + health.fa + inequality.fa | region),data = filter(afc.data,Type == "textbook",Ju
```

```
## Warning: Using formula(x) is deprecated when x is a character vector of length > 1.
##   Consider formula(paste(x, collapse = " ")) instead.
```

```
summary(model21) %>% short(title = "Ecological Factors and Femininity 3afc - Textbook Fertility")
```

Table 66: Ecological Factors and Femininity 3afc - Textbook Fertility

	Estimate	Std. Error	z value	p value
-0.5|0	-0.544	0.168	-3.227	0.001
0|0.5	0.830	0.170	4.879	0.000
health.fa	-0.208	0.123	-1.692	0.091
inequality.fa	-0.033	0.080	-0.417	0.676

Rating

```
model_a6 <- lmer(rating ~ health.fa*level + inequality.fa*level +
                  (1 + level || country_name) +
                  (1 + health.fa:level + inequality.fa:level || region) +
                  (1 + health.fa:level + inequality.fa:level || user_id),data = filter(rating.data,Type
```

```
## boundary (singular) fit: see ?isSingular
```

```
## Warning: Model failed to converge with 1 negative eigenvalue: -8.5e+01
```

```
summary(model_a6) %>% short(title = "Ecological Factors and Attractiveness Ratings - Textbook Fertility
```

```
## Warning in if (class(s) == "summary.clmm") {: the condition has length > 1 and
## only the first element will be used
```

Table 67: Ecological Factors and Attractiveness Ratings - Textbook Fertility

	Estimate	Std. Error	df	t value	p value
(Intercept)	4.434	0.116	8.892	38.157	0.000
health.fa	-0.045	0.117	7.786	-0.382	0.713
level	-0.011	0.056	1719.296	-0.202	0.840
inequality.fa	-0.087	0.112	5.891	-0.779	0.466
health.fa:level	-0.062	0.062	1589.030	-1.006	0.315
level:inequality.fa	-0.051	0.039	1979.815	-1.292	0.197

```
model_a21 <- lmer(rating ~ health.fa*level + inequality.fa*level +
                  (1 + level || country_name) +
                  (1 + health.fa:level + inequality.fa:level || region) +
                  (1 + health.fa:level + inequality.fa:level || user_id),data = filter(rating.data,Type
```

```
## boundary (singular) fit: see ?isSingular
```

```
summary(model_a21) %>% short(title = "Ecological Factors and Femininity Ratings - Textbook Fertility")
```

```
## Warning in if (class(s) == "summary.clmm") {: the condition has length > 1 and
## only the first element will be used
```

Table 68: Ecological Factors and Femininity Ratings - Textbook Fertility

	Estimate	Std. Error	df	t value	p value
(Intercept)	4.716	0.137	2.062	34.369	0.001
health.fa	0.019	0.135	6.406	0.143	0.891
level	-0.013	0.061	78.270	-0.207	0.836
inequality.fa	-0.069	0.132	4.636	-0.524	0.625
health.fa:level	-0.038	0.067	53.215	-0.564	0.575
level:inequality.fa	-0.021	0.087	1.480	-0.244	0.837

E2

3afc

```
model9 <- clmm(as.factor(choice) ~ health.fa + inequality.fa +
                  (1 | country_name) +
                  (1 + health.fa + inequality.fa | region),data = filter(afc.data,Type == "E2",Judgemen
```

```
## Warning: Using formula(x) is deprecated when x is a character vector of length > 1.
##   Consider formula(paste(x, collapse = " ")) instead.
```

```
summary(model9) %>% short(title = "Ecological Factors and Attractiveness 3afc - Levels of E")
```

Table 69: Ecological Factors and Attractiveness 3afc - Levels of E

	Estimate	Std. Error	z value	p value
-0.5|0	-0.728	0.099	-7.335	0.000
0|0.5	0.603	0.098	6.135	0.000
health.fa	-0.076	0.100	-0.761	0.447
inequality.fa	0.120	0.061	1.970	0.049

```
model24 <- clmm(as.factor(choice) ~ health.fa + inequality.fa +
                  (1 | country_name) +
                  (1 + health.fa + inequality.fa | region),data = filter(afc.data,Type == "E2",Judgemen
```

```
## Warning: Using formula(x) is deprecated when x is a character vector of length > 1.
##   Consider formula(paste(x, collapse = " ")) instead.
```

```
summary(model24) %>% short(title = "Ecological Factors and Femininity 3afc - Levels of E")
```

Table 70: Ecological Factors and Femininity 3afc - Levels of E

	Estimate	Std. Error	z value	p value
-0.5|0	-0.684	0.098	-6.962	0.000
0|0.5	0.721	0.099	7.317	0.000
health.fa	0.050	0.099	0.506	0.613
inequality.fa	0.028	0.061	0.456	0.648

Rating

```
model_a9 <- lmer(rating ~ health.fa*level + inequality.fa*level +
                  (1 + level || country_name) +
                  (1 + health.fa:level + inequality.fa:level || region) +
                  (1 + health.fa:level + inequality.fa:level || user_id),data = filter(rating.data,Type
```

```
## boundary (singular) fit: see ?isSingular
```

```
summary(model_a9) %>% short(title = "Ecological Factors and Attractiveness Ratings - Levels of E")
```

```
## Warning in if (class(s) == "summary.clmm") {: the condition has length > 1 and
## only the first element will be used
```

Table 71: Ecological Factors and Attractiveness Ratings - Levels of E

	Estimate	Std. Error	df	t value	p value
(Intercept)	4.333	0.108	9.470	40.244	0.000
health.fa	-0.054	0.108	8.490	-0.498	0.631
level	0.125	0.047	452.439	2.671	0.008
inequality.fa	-0.053	0.101	5.836	-0.526	0.618
health.fa:level	-0.008	0.052	404.205	-0.159	0.874
level:inequality.fa	0.008	0.034	797.799	0.236	0.813

```
model_a24 <- lmer(rating ~ health.fa*level + inequality.fa*level +
                  (1 + level || country_name) +
                  (1 + health.fa:level + inequality.fa:level || region) +
                  (1 + health.fa:level + inequality.fa:level || user_id),data = filter(rating.data,Type
```

```
## boundary (singular) fit: see ?isSingular
```

```
## Warning: Model failed to converge with 1 negative eigenvalue: -5.6e+02
```

```
summary(model_a24) %>% short(title = "Ecological Factors and Femininity Ratings - Levels of E")
```

```
## Warning in if (class(s) == "summary.clmm") {: the condition has length > 1 and
## only the first element will be used
```

Table 72: Ecological Factors and Femininity Ratings - Levels of E

	Estimate	Std. Error	df	t value	p value
(Intercept)	4.709	0.134	7.505	35.173	0.000
health.fa	-0.034	0.134	6.453	-0.255	0.806
level	0.064	0.048	447.676	1.352	0.177

	Estimate	Std. Error	df	t value	p value
inequality.fa	-0.013	0.130	5.138	-0.097	0.926
health.fa:level	0.019	0.052	396.150	0.370	0.712
level:inequality.fa	-0.019	0.035	823.720	-0.529	0.597

P

3afc

```
model12 <- clmm(as.factor(choice) ~ health.fa + inequality.fa +
                  (1 | country_name) +
                  (1 + health.fa + inequality.fa | region),data = filter(afc.data,Type == "P",Judgement
```

```
## Warning: Using formula(x) is deprecated when x is a character vector of length > 1.
##   Consider formula(paste(x, collapse = " ")) instead.
```

```
summary(model12) %>% short(title = "Ecological Factors and Attractiveness 3afc - Levels of P")
```

Table 73: Ecological Factors and Attractiveness 3afc - Levels of P

	Estimate	Std. Error	z value	p value
-0.5|0	-0.621	0.135	-4.599	0.000
0|0.5	0.829	0.138	5.990	0.000
health.fa	-0.062	0.112	-0.551	0.581
inequality.fa	-0.018	0.068	-0.263	0.793

```
model27 <- clmm(as.factor(choice) ~ health.fa + inequality.fa +
                  (1 | country_name) +
                  (1 + health.fa + inequality.fa | region),data = filter(afc.data,Type == "P",Judgement
```

```
## Warning: Using formula(x) is deprecated when x is a character vector of length > 1.
##   Consider formula(paste(x, collapse = " ")) instead.
```

```
summary(model27) %>% short(title = "Ecological Factors and Femininity 3afc - Levels of P")
```

```
## Warning in summary.clmm(model27): Variance-covariance matrix of the parameters
## is not defined
```

Table 74: Ecological Factors and Femininity 3afc - Levels of P

	Estimate	Std. Error	z value	p value
-0.5|0	-0.621	NaN	NaN	NaN
0|0.5	0.786	NaN	NaN	NaN
health.fa	-0.104	NaN	NaN	NaN
inequality.fa	-0.069	NaN	NaN	NaN

Rating

```
model_a12 <- lmer(rating ~ health.fa*level + inequality.fa*level +
                  (1 + level || country_name) +
                  (1 + health.fa:level + inequality.fa:level || region) +
                  (1 + health.fa:level + inequality.fa:level || user_id),data = filter(rating.data,Type
```

```
## boundary (singular) fit: see ?isSingular
```

```r
summary(model_a12) %>% short(title = "Ecological Factors and Attractiveness Ratings - Levels of P")
```

```
## Warning in if (class(s) == "summary.clmm") {: the condition has length > 1 and
## only the first element will be used
```

Table 75: Ecological Factors and Attractiveness Ratings - Levels of P

	Estimate	Std. Error	df	t value	p value
(Intercept)	4.338	0.098	9.600	44.294	0.000
health.fa	-0.079	0.098	9.005	-0.812	0.438
level	0.031	0.061	7.510	0.502	0.630
inequality.fa	-0.004	0.090	5.470	-0.049	0.962
health.fa:level	-0.014	0.062	12.880	-0.232	0.821
level:inequality.fa	-0.021	0.050	3.323	-0.413	0.705

```r
model_a27 <- lmer(rating ~ health.fa*level + inequality.fa*level +
                  (1 + level || country_name) +
                  (1 + health.fa:level + inequality.fa:level || region) +
                  (1 + health.fa:level + inequality.fa:level || user_id),data = filter(rating.data,Type
```

```
## boundary (singular) fit: see ?isSingular
```

```
## Warning: Model failed to converge with 1 negative eigenvalue: -5.2e+02
```

```r
summary(model_a27) %>% short(title = "Ecological Factors and Femininity Ratings - Levels of P")
```

```
## Warning in if (class(s) == "summary.clmm") {: the condition has length > 1 and
## only the first element will be used
```

Table 76: Ecological Factors and Femininity Ratings - Levels of P

	Estimate	Std. Error	df	t value	p value
(Intercept)	4.647	0.121	9.005	38.339	0.000
health.fa	-0.036	0.121	7.969	-0.301	0.771
level	-0.115	0.055	449.928	-2.106	0.036
inequality.fa	-0.006	0.114	5.553	-0.053	0.960
health.fa:level	-0.095	0.061	409.392	-1.564	0.119
level:inequality.fa	-0.033	0.039	758.351	-0.863	0.388

EtoP

3afc

```r
model15 <- clmm(as.factor(choice) ~ health.fa + inequality.fa +
                  (1 | country_name) +
                  (1 + health.fa + inequality.fa | region),data = filter(afc.data,Type == "EtoP",Judgeme
```

```
## Warning: Using formula(x) is deprecated when x is a character vector of length > 1.
##   Consider formula(paste(x, collapse = " ")) instead.
```

```
summary(model15) %>% short(title = "Ecological Factors and Attractiveness 3afc - E to P Ratio")
```

Table 77: Ecological Factors and Attractiveness 3afc - E to P Ratio

	Estimate	Std. Error	z value	p value
-0.5|0	-0.511	0.098	-5.188	0.000
0|0.5	0.757	0.100	7.567	0.000
health.fa	0.084	0.106	0.796	0.426
inequality.fa	-0.054	0.064	-0.835	0.403

```
model30 <- clmm(as.factor(choice) ~ health.fa + inequality.fa +
                  (1 | country_name) +
                  (1 + health.fa + inequality.fa | region),data = filter(afc.data,Type == "EtoP",Judgeme
```

```
## Warning: Using formula(x) is deprecated when x is a character vector of length > 1.
##   Consider formula(paste(x, collapse = " ")) instead.
```

```
summary(model30) %>% short(title = "Ecological Factors and Femininity 3afc - E to P Ratio")
```

Table 78: Ecological Factors and Femininity 3afc - E to P Ratio

	Estimate	Std. Error	z value	p value
-0.5|0	-0.613	0.100	-6.109	0.000
0|0.5	0.666	0.101	6.623	0.000
health.fa	0.020	0.103	0.195	0.846
inequality.fa	0.082	0.062	1.315	0.189

Rating

```
model_a15 <- lmer(rating ~ health.fa*level + inequality.fa*level +
                  (1 + level || country_name) +
                  (1 + health.fa:level + inequality.fa:level || region) +
                  (1 + health.fa:level + inequality.fa:level || user_id),data = filter(rating.data,Type
```

```
## boundary (singular) fit: see ?isSingular
```

```
summary(model_a15) %>% short(title = "Ecological Factors and Attractiveness Ratings - E to P Ratio")
```

```
## Warning in if (class(s) == "summary.clmm") {: the condition has length > 1 and
## only the first element will be used
```

Table 79: Ecological Factors and Attractiveness Ratings - E to P Ratio

	Estimate	Std. Error	df	t value	p value
(Intercept)	4.425	0.109	8.924	40.562	0.000
health.fa	0.011	0.109	8.013	0.105	0.919
level	0.054	0.048	452.442	1.135	0.257
inequality.fa	-0.016	0.103	5.478	-0.160	0.879
health.fa:level	0.012	0.053	400.875	0.235	0.814
level:inequality.fa	0.002	0.035	818.376	0.052	0.959

```r
model_a30 <- lmer(rating ~ health.fa*level + inequality.fa*level +
                  (1 + level || country_name) +
                  (1 + health.fa:level + inequality.fa:level || region) +
                  (1 + health.fa:level + inequality.fa:level || user_id),data = filter(rating.data,Type
```

```
## boundary (singular) fit: see ?isSingular
```

```
## Warning: Model failed to converge with 1 negative eigenvalue: -2.4e+02
```

```r
summary(model_a30) %>% short(title = "Ecological Factors and Femininity Ratings - E to P Ratio")
```

```
## Warning in if (class(s) == "summary.clmm") {: the condition has length > 1 and
## only the first element will be used
```

Table 80: Ecological Factors and Femininity Ratings - E to P Ratio

	Estimate	Std. Error	df	t value	p value
(Intercept)	4.684	0.148	7.986	31.686	0.000
health.fa	0.007	0.150	6.863	0.045	0.965
level	0.050	0.067	4.362	0.747	0.493
inequality.fa	-0.028	0.146	5.881	-0.195	0.852
health.fa:level	-0.001	0.067	6.784	-0.008	0.994
level:inequality.fa	0.056	0.055	2.033	1.027	0.411
